# Biofilm formation of *Pseudomonas aeruginosa* in spaceflight is minimized on lubricant impregnated surfaces

**DOI:** 10.1038/s41526-023-00316-w

**Published:** 2023-08-16

**Authors:** Pamela Flores, Samantha A. McBride, Jonathan M. Galazka, Kripa K. Varanasi, Luis Zea

**Affiliations:** 1 BioServe Space Technologies, Aerospace Engineering Sciences Department, University of Colorado Boulder, Boulder, CO 80309 USA; 2 Molecular, Cellular, and Developmental Biology Department, University of Colorado Boulder, Boulder, CO 80309 USA; 3 Massachusetts Institute of Technology (MIT), Cambridge, MA 02139 USA; 4 Space Biosciences Division, NASA Ames Research Center, Moffett Field, CA 94035 USA

**Keywords:** Microbiology, Molecular biology, Aerospace engineering

## Abstract

The undesirable, yet inevitable, presence of bacterial biofilms in spacecraft poses a risk to the proper functioning of systems and to astronauts’ health. To mitigate the risks that arise from them, it is important to understand biofilms’ behavior in microgravity. As part of the Space Biofilms project, biofilms of *Pseudomonas aeruginosa* were grown in spaceflight over material surfaces. Stainless Steel 316 (SS316) and passivated SS316 were tested for their relevance as spaceflight hardware components, while a lubricant impregnated surface (LIS) was tested as potential biofilm control strategy. The morphology and gene expression of biofilms were characterized. Biofilms in microgravity are less robust than on Earth. LIS strongly inhibits biofilm formation compared to SS. Furthermore, this effect is even greater in spaceflight than on Earth, making LIS a promising option for spacecraft use. Transcriptomic profiles for the different conditions are presented, and potential mechanisms of biofilm reduction on LIS are discussed.

## Introduction

In previous and current spaces stations (Salyut-6 and -7, Mir, and the International Space Station (ISS)), damage caused by bacterial biofilms on hardware have resulted in function disruption and failures of myriad equipment (summarized in Zea et al., 2018, 2020^1,2^). On the ISS, biofilms are a concern for the water recovery system, particularly for the water processing assembly’s wastewater tank and its downstream filter^3,4^, as well as for the hoses linking the distillation assembly to the purge and fluid pumps, which have needed to be returned to Earth for unclogging and reprocessing before sent back to ISS (Y.A. Velez-Justiniano, personal communication, June 10, 2022).

Biofilms are communities of microbes attached to each other and to surfaces which can cause damage to materials either directly by using it as food, or indirectly via their metabolic byproducts^5,6^. While pre-planned biofilms may enable useful processes in space^7–9^, uncontrolled growth—especially of opportunistic or pathogenic microbes—may have another deleterious effect for space missions: increased risk of infections to the crew. Microbial communities living as biofilms can have increased tolerance to disinfectants and antibiotics^10^, which may contribute to recurrent infections. On Earth, biofilms are associated with 65% and 80% of infectious and chronic diseases, respectively^11^.

Materials can profoundly influence biofilm growth, and it is possible to design surfaces that either prevent microbial adhesion or that have antimicrobial properties. Antimicrobial surfaces include those with metallic nanoparticles with biocidal effects^12^ and strongly oxidizing materials that destroy cell membranes^13^. However, biocidal surfaces have a critical failure mechanism when a layer of destroyed cells forms on the initial surface. This layer can provide a safe harbor for new microbes to colonize. Preventing initial adhesion of microbes via engineering of surface properties addresses this limitation. Surface roughness provides initial colonizers with favorable positions to adhere to a surface, and therefore smooth surfaces tend to be more successful at preventing adhesion^14^. Hydrophobicity is another property that is particularly focused on in the context of creating adhesion-free surfaces; however, the influence is a complicated one. In general, hydrophobic surfaces may cause greater initial attachment due to hydrophobic interactions between the surface and the cell membranes, but also allow larger detachment rates^14^.

The fast pace of microbial adaptation and changes in gene expression also presents a significant challenge for creation of anti-biofouling materials. Microorganism behavior is significantly altered by the presence of different surfaces. For example, biofilm morphologies differ strongly across steel and polypropylene surfaces^15^, and gene expression is altered for microbes exposed to less habitable surfaces^16,17^. Therefore, even surfaces engineered to resist biofilms may not be immune to bacteria colonization.

Biofilm growth in space stations and surface habitat components can decrease the probability of mission success and increase that of medical risks to the crew. Crewed missions to the Lunar surface and Mars, the latter requiring several years in space, warrant understanding of how biofilms grow differently in microgravity, as system functionality is of even higher importance when receiving spare parts from Earth or returning crew to Earth promptly is not possible. To address this, the NASA-funded Space Biofilms project was designed as described in Zea et al. 2018^1^, and prepared and performed on board the ISS as reported in Flores et al., 2022^18^. In brief, samples of *Pseudomonas aeruginosa* UCBPP-PA14 (PA14) were sent to the ISS and biofilms were allowed to grow on different surfaces for either one, two, or three days at 37 °C. Equivalent biofilms grown on Earth at 1 *g* were used as controls. The six material surfaces tested were stainless steel 316 (SS316), passivated SS316 (pSS316), a silicon wafer impregnated with silicone oil as a lubricant-impregnated surface (LIS)^19^, cellulose membrane, silicone, and silicone with a microtopography. Each condition tested had four replicates fixed in 4% PFA for morphology analysis, and other four preserved in RNAlater for gene expression analysis. Here, we report the morphological and transcriptomic results of SS316, pSS316, and LIS grown in nutrient-rich medium (to replicate wastewater); the rest are reported elsewhere^20^.

SS316 and pSS316 were assessed given their common use for spacecraft components and for elements of the Environmental Control and Life Support Systems (ECLSS), respectively. LIS was tested as a potential alternative for future spacecraft parts most susceptible to biofilm formation.

**Fig. 1:**
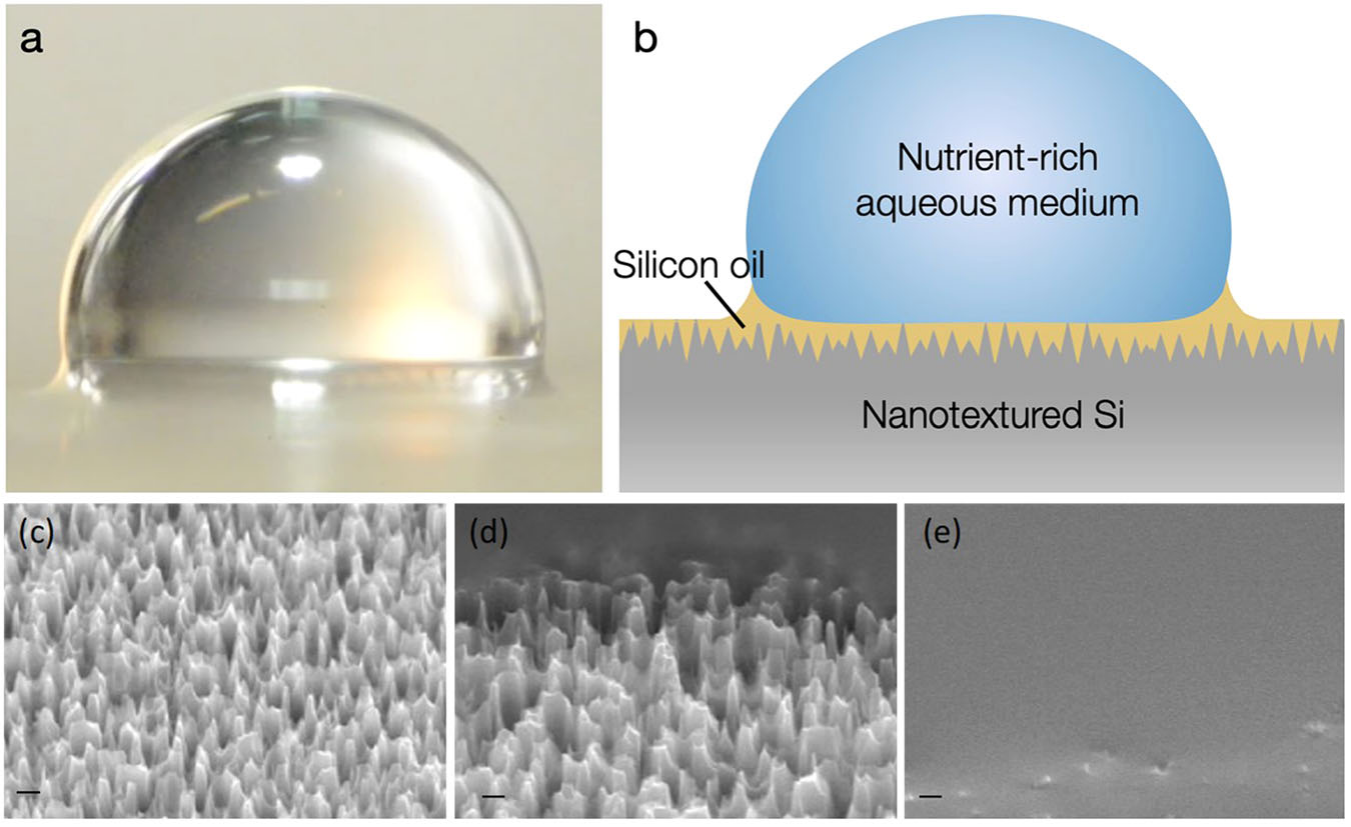
Liquid impregnated surfaces. **a** Optical image of drop of water on LIS, **b** Cartoon showing different components of LIS, **c**–**e** representative SEM images of nanotextured silicon wafer (nanograss) **c** dry, **d** lubricant wicking into nanograss, and **e** nanograss stably impregnated by lubricant. Scale bars are 400 nm.

SS316 is an iron alloy containing chromium, nickel, and molybdenum characteristic for its increased resistance to corrosion compared to iron^21^. The passivation of SS316 uses a nitric or citric acid treatment to eliminate iron from the surface and generate an oxide passive layer to prevent corrosion^22^. LISs are a class of textured surfaces that are stably impregnated with a lubricant (e.g., oils) to render the surface with super-slippery properties^19,23^ (Fig. 1). The lubricant is held within the texture by capillary and intermolecular forces. A drop on LIS can exist in one of twelve different thermodynamic states depending on the properties of the solid texture, lubricant, droplet, and environment. Stable impregnation of the lubricant requires the contact angle of the lubricant on solid surface to be below a critical value set by the texture parameters. For the case of stable LIS, the texture tops can remain exposed or covered by a thin van der Waals lubricant film depending on the spreading coefficient of the lubricant on the solid surface (in the presence of droplet phase). A detailed discussion of the thermodynamic states, stable impregnation criteria, and droplet mobility are provided in Smith et al. (2013)^19^. Because a significant portion of the surface texture can stay submerged in the lubricant (except for texture tops) for stable LIS, these surfaces can achieve significant reduction in adhesion and have been shown to have remarkable antifouling properties^24–30^.

## Results

The biomass, thickness, and surface area coverage of biofilms formed on SS316 were not statistically different from those of biofilms grown on pSS316 (*α* = 0.05) at any incubation day (Supplementary Figure 1 and raw data in Supplementary Table 1). Similarly, no morphological differences were observed between biofilms formed on the two materials (Supplementary Figure 2) for either Earth-based or microgravity cultures. In addition, there were no differentially expressed genes (DEG) between SS316 and pSS316 biofilms in any conditions tested (*α* = 0.05) except for microgravity day 2 where pSS316 had 44 DEG (Supplementary Table 2). Based on this, SS316 and pSS316 were pooled into one “SS” data set which is hereon compared and contrasted to that of biofilms formed on LIS.

### Biofilm morphology and observations from the liquid cultures

**Fig. 2:**
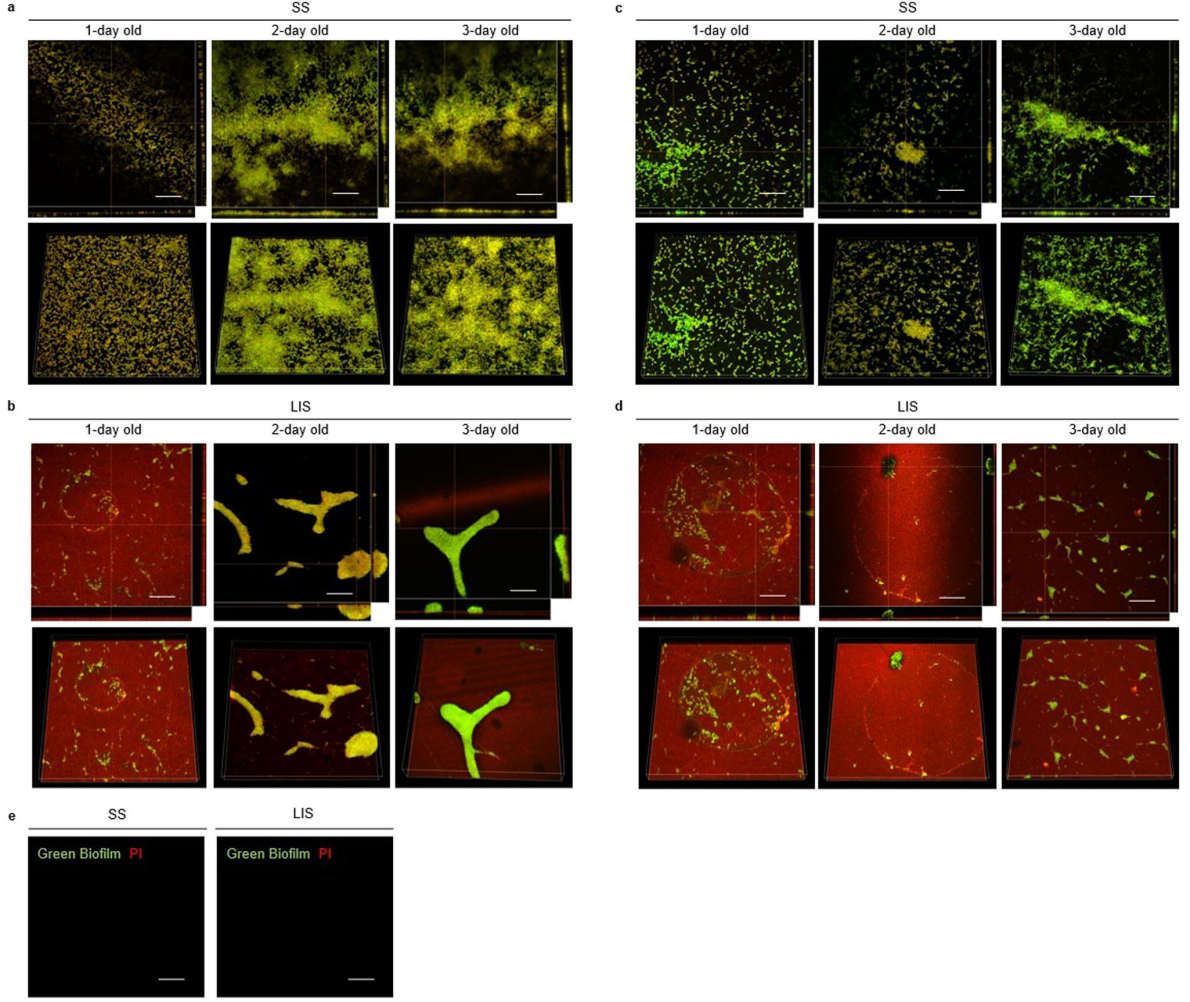
*P. aeruginosa* biofilm morphology on SS and LIS in 1 *g* or microgravity. Representative confocal microscopy images of 1-, 2-, and 3-day-old biofilms formed over SS and LIS. Nucleic acids were stained with PI (red) and lipids were stained with Green Biofilm (green). First row per material is a bottom slice image with side-view panels of a cross-section point in the biofilm (specific section marked by the orange lines). Second row per material is a volume view of the complete z-stack image. Images correspond to biofilms grown in 1 *g* over **a** SS and **b** LIS, or for biofilms grown in microgravity over **c** SS and **d** LIS. Negative controls of the material surfaces, stained with both PI and Green Biofilm stains, are presented in panel (**e**). Scale bars are 20 µm. Biofilms form without boundaries on SS while on LIS they have sharp edges. Biofilms had significantly reduced surface area coverage on LIS, especially in microgravity where biofilms were minimized and mostly present as small groups of cells. The thin layer of nucleic acids on LIS surface could be DNA, RNA, or both as PI does not differentiate between them.

In 1 *g*, biofilms on SS grew throughout the surface with no marked boundaries, usually creating conglomerates that formed ‘mounts’ of biofilm with dispersed single cells in between (Fig. 2a). Conversely, the few biofilms grown on LIS were restricted to certain areas with sharp edges, had a more compact appearance, and looked thicker (not significant) than those grown on SS (Fig. 2b). In addition, the LIS surface showed individual cells or small multiple cell groups attached in random isolated areas and a thin layer of nucleic acids in the areas where there was no biofilm. Bacterial cells attached on LIS were often found growing in the perimeter or inside of a circle (as seen for 1-day-old biofilms on 1 *g*) as well as small, connected clusters. Overall, biofilms on LIS seemed to be restricted to grow in smaller areas than on SS.

The morphology of biofilms formed on SS in space was similar to that of the Earth controls, in that the space samples also exhibited a lack of clear boundaries and biofilm ‘mounts’ were also observed, albeit with bigger gaps between them (Fig. 2c). Biofilms grown on LIS in microgravity did not form as many sharp, compact, and thick biofilms (only a few in smaller size) compared to those formed in 1 *g* (Fig. 2d). The thickness of these smaller biofilms decreased in time (*p* < 0.001). Most of the surface of LIS was covered with a thin layer of nucleic acids (red signal from the propidium iodide (PI) stain). The PI did not partition to LIS’s silicone oil in the absence of bacteria, nor did the Green Biofilm stain (green signal to label lipids) (Fig. 2e) confirming that the signal on the biofilm samples corresponds to adsorbed nucleic acids on LIS’s surface.

The PA14 liquid cultures in which the coupons were submerged became more turbid (turbidity correlation with viable cell count available in Supplementary Figure 3) for the microgravity set than for the 1 *g* controls (not significant for SS day 1 and SS day 2). Specifically, for the samples cultured for three days, when OD_600_ in microgravity had an increase of 41.3% for SS (*p* = 2.9e−08) and 23.4% for LIS (*p* = 0.0204) (Supplementary Figure 4 and raw data in Supplementary Table 3). In addition, it is worth noting that the color of the liquid culture changed as well. In microgravity, the cultures were light green on day 1, light brown on day 2, and light pink on day 3, while the ground cultures remained clear for days 1 and 2 and then turned light yellow on day 3 (Supplementary Figure 5). The increased culture turbidity in microgravity could suggest more bacterial growth in general; however, microgravity biofilms exhibited the opposite trend.

### Biofilm biomass, thickness, and surface coverage

**Fig. 3:**
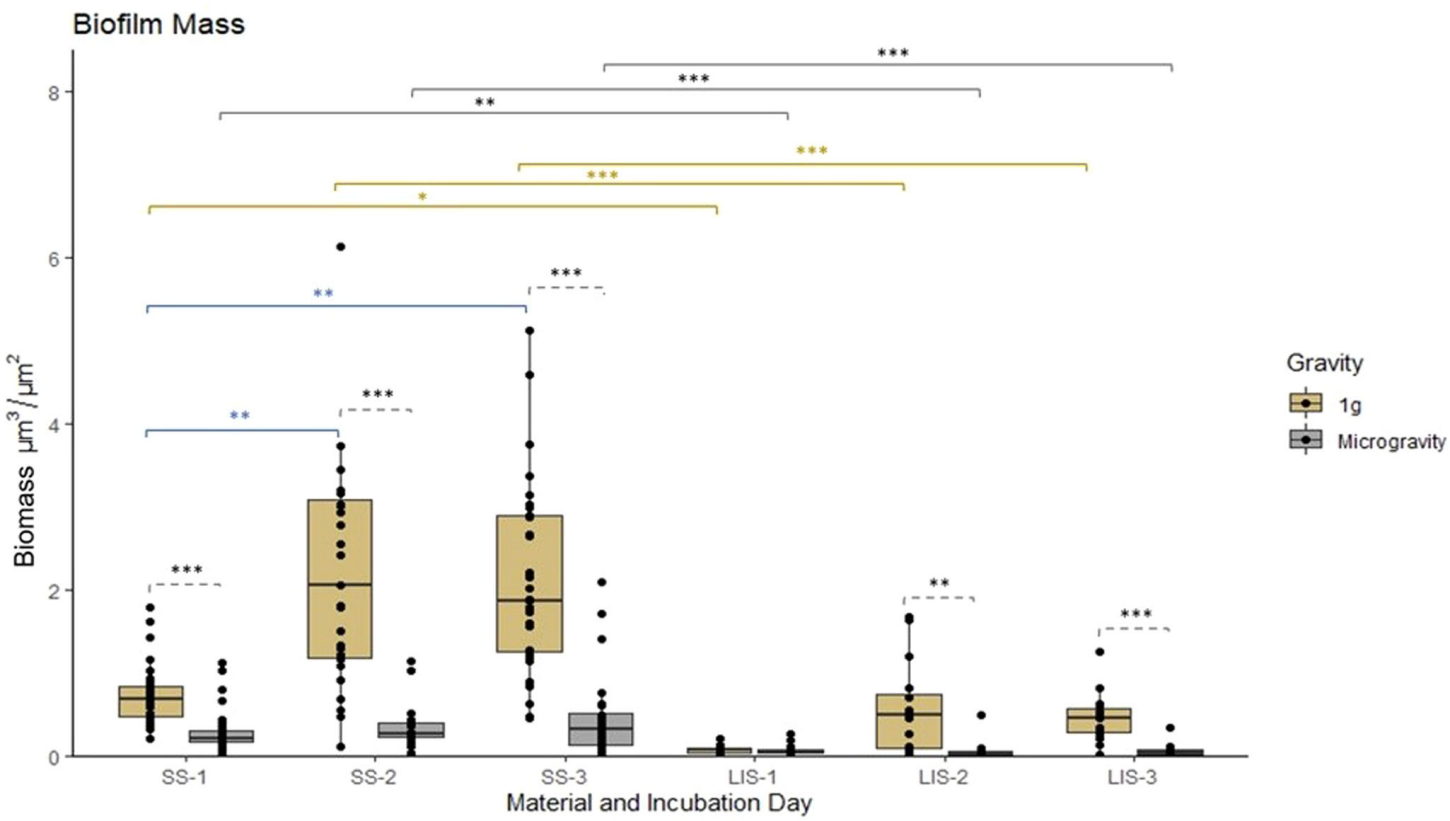
*P. aeruginosa* biofilm mass on SS and LIS in 1 *g* or microgravity. Biofilm mass plotted as a function of time (1-, 2-, and 3-day-old biofilms) and gravitational condition. Statistical significance specified with horizontal brackets, for comparisons between microgravity and 1 *g* (dashed brackets), for comparisons of SS vs LIS in 1 *g* (gold brackets) or in microgravity (gray brackets), and for comparisons of SS between time points (blue brackets). SS= stainless steel. LIS = lubricant impregnated surface. For SS, *n* = 8 biological replicates each imaged in 4 fields of view. For LIS, *n* = 4 biological replicates each imaged in 4 fields of view. Dunn’s test with Bonferroni correction ***p ≤ 0.001, **p ≤ 0.01, *p ≤ 0.05. Box central line= median, bounds of box=first and third quartile, whiskers=minimum and maximum values (without outliers).

**Fig. 4:**
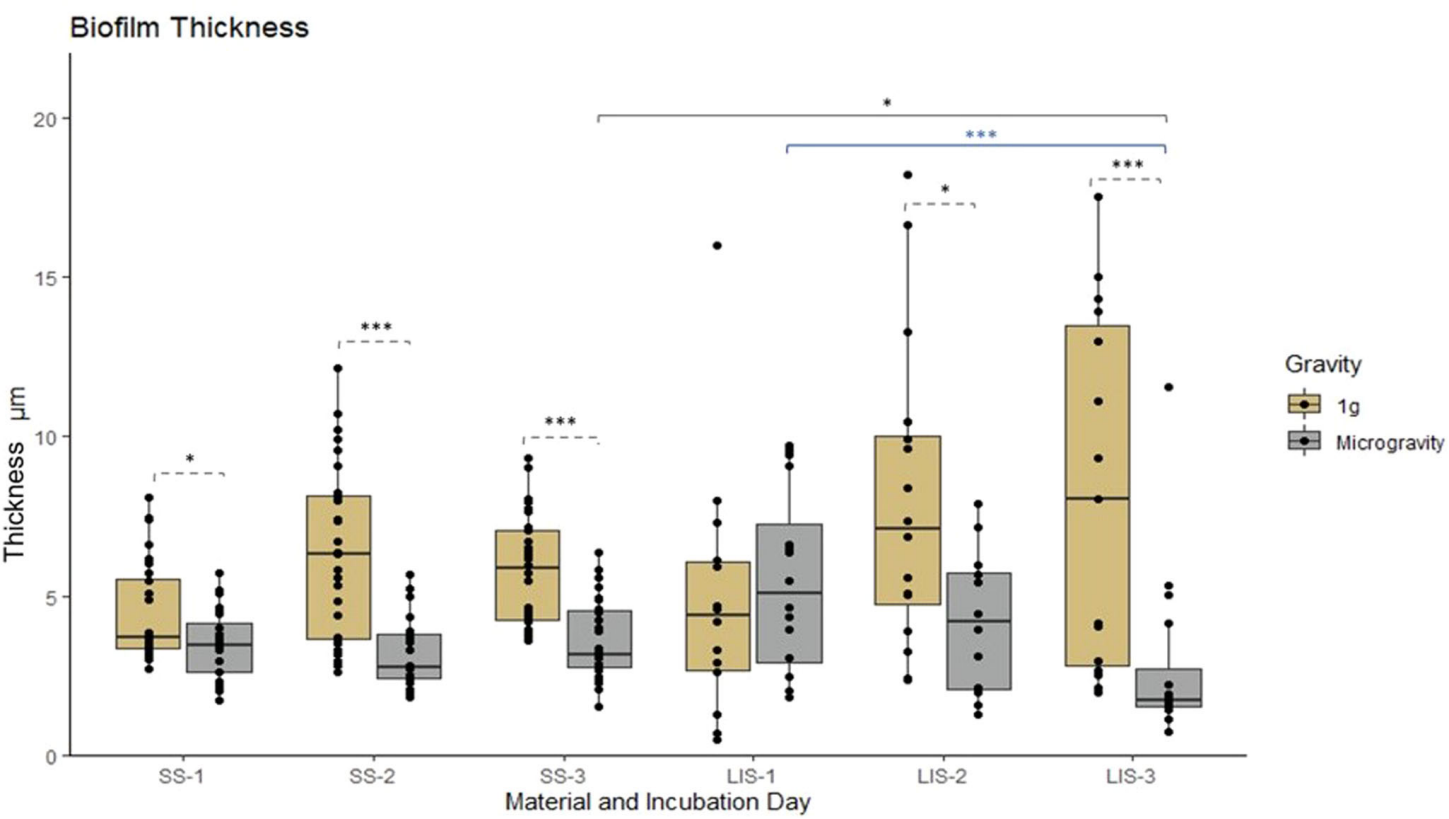
*P. aeruginosa* biofilm thickness on SS and LIS in 1 *g* or microgravity. Biofilm thickness plotted as a function of time (1-, 2-, and 3-day-old biofilms) and gravitational condition. Statistical significance specified with horizontal brackets, for comparisons between microgravity and 1 *g* (dashed brackets), for comparisons of SS vs LIS in microgravity (gray brackets), and for comparisons of LIS between time points (blue brackets). SS = stainless steel. LIS = lubricant impregnated material. For SS, *n* = 8 biological replicates each imaged in 4 fields of view. For LIS, *n* = 4 biological replicates each imaged in 4 fields of view. Dunn’s test with Bonferroni correction ***p ≤ 0.001, **p ≤ 0.01, *p ≤ 0.05. Box central line = median, bounds of box = first and third quartile, whiskers = minimum and maximum values (without outliers).

**Fig. 5:**
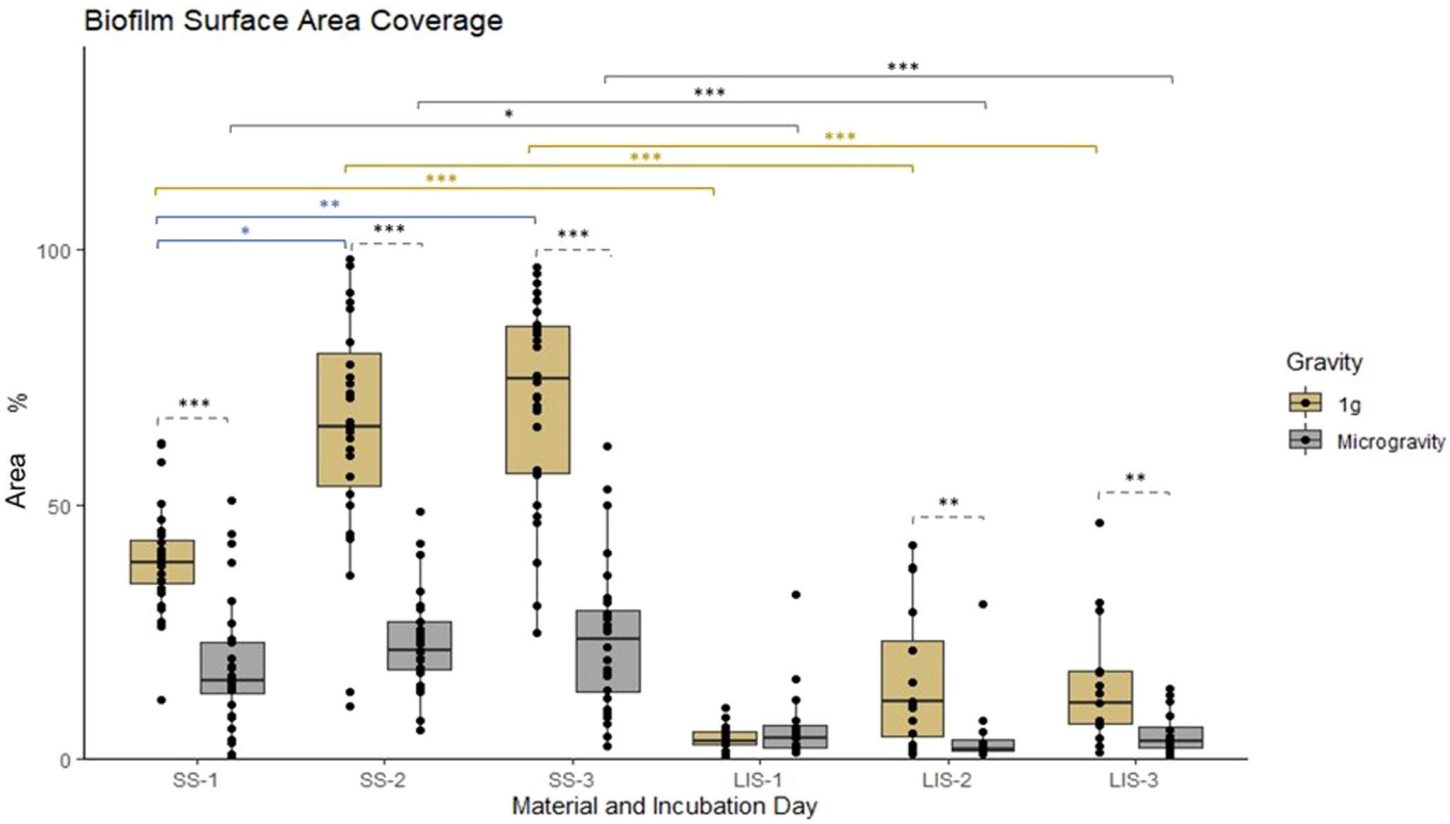
*P. aeruginosa* biofilm surface area coverage on SS and LIS in 1 *g* or microgravity. The percentage of the imaged surface covered with biofilm plotted as a function of time (1-, 2-, and 3-day old biofilms) and gravitational condition. Statistical significance specified with horizontal brackets, for comparisons between microgravity and 1 *g* (dashed brackets), for SS vs LIS in 1 *g* (gold brackets) or in microgravity (gray brackets), and for SS between time points (blue brackets). SS = stainless steel. LIS = lubricant impregnated material. For SS, *n* = 8 biological replicates each imaged in 4 fields of view. For LIS, *n* = 4 biological replicates each imaged in 4 fields of view. Dunn’s test with Bonferroni correction ***p ≤ 0.001, **p ≤ 0.01, *p ≤ 0.05. Box central line = median, bounds of box = first and third quartile, whiskers=minimum and maximum values (without outliers).

PA14 biofilms formed in microgravity had significantly lower (*p* ≤ 0.05) biomass (Fig. 3), and thickness (Fig. 4) than those formed at 1 *g*. This is true for both SS and LIS, the only exception being for the biofilms formed on LIS at one day of incubation, which exhibited no difference between gravitational regimes. In the case of SS, by day 3 biofilms formed in microgravity had only 17.6% and 53.6% of the ground control biomass and thickness, respectively. The same phenomenon was observed on LIS, where biofilms formed in space had 8.7% and 21.3% of the ground control biomass and thickness. Furthermore, biofilms formed in space covered significantly less surface than their 1 *g* counterparts (31.4% and 33.0% of surface area for SS and LIS, respectively) (Fig. 5). The median values for the three parameters assessed are summarized in Table 1 and the raw data in Supplementary Table 4.

**Table 1:** Median values of biofilm biomass, thickness, and surface area coverage per day and material.

Material & incubation time	Biomass (µm^3^/µm^2^) 1 *g*	Biomass (µm^3^/µm^2^) µg	Thickness (µm) 1 *g*	Thickness (µm) µg	Surface area coverage (%) 1 *g*	Surface area coverage (%) µg
SS day 1	0.69 (0.38)	0.22 (0.13)	3.70 (2.19)	3.44 (1.52)	38.76 (8.57)	15.48 (10.24)
SS day 2	2.06 (1.90)	0.27 (0.18)	6.30 (4.53)	2.77 (1.38)	65.38 (25.77)	21.19 (9.48)
SS day 3	1.87 (1.64)	0.33 (0.38)	5.86 (2.82)	3.14 (1.76)	74.79 (28.97)	23.49 (15.93)
LIS day 1	0.07 (0.06)	0.05 (0.05)	4.41 (3.41)	5.06 (4.32)	3.40 (2.55)	4.20 (4.30)
LIS day 2	0.49 (0.64)	0.02 (0.04)	7.09 (5.29)	4.21 (3.66)	11.17 (18.79)	2.00 (2.22)
LIS day 3	0.46 (0.28)	0.04 (0.05)	8.04 (10.63)	1.71 (1.18)	10.87 (10.21)	3.60 (4.30)

Results are presented as median and the interquartile range Q3-Q1 is indicated in parenthesis. For SS, *n* = 8 biological replicates each imaged in 4 fields of view. For LIS, *n* = 4 biological replicates each imaged in 4 fields of view.

*SS* stainless steel, *LIS* lubricant impregnated surface.

The SS and LIS biofilms on 1 *g* appeared to grow in size with time, with the largest growth observed between day 1 and day 2 (Figs. 2a,b, 3, 4, 5). This trend also held true for SS in microgravity; although with a lower growth rate (Figs. 2c, 3, 4, 5). For biofilms on LIS in microgravity, there was little to no change in mass and surface area coverage as a function of time.

Biofilms grown on SS were significantly different than biofilms grown on LIS in terms of mass and surface area coverage. On both Earth and in microgravity, at day 3, the median of biomass and surface area coverage in LIS was significantly lower (*p* ≤ 0.001) than in SS biofilms (Table 1).

### Gene expression of biofilms in microgravity vs biofilms on 1 *g*

Bacteria adapt to their environment through gene expression modulation. The mechanisms used by bacteria to adapt to changes can be observed in their transcriptomic profile. The following paragraphs compare 3-day-old biofilms formed in microgravity against matched Earth controls (baseline) for either SS or LIS surfaces. When available, the PAO1 ortholog gene numbers are listed in the brackets at the first mention of the corresponding PA14 gene.

**Fig. 6:**
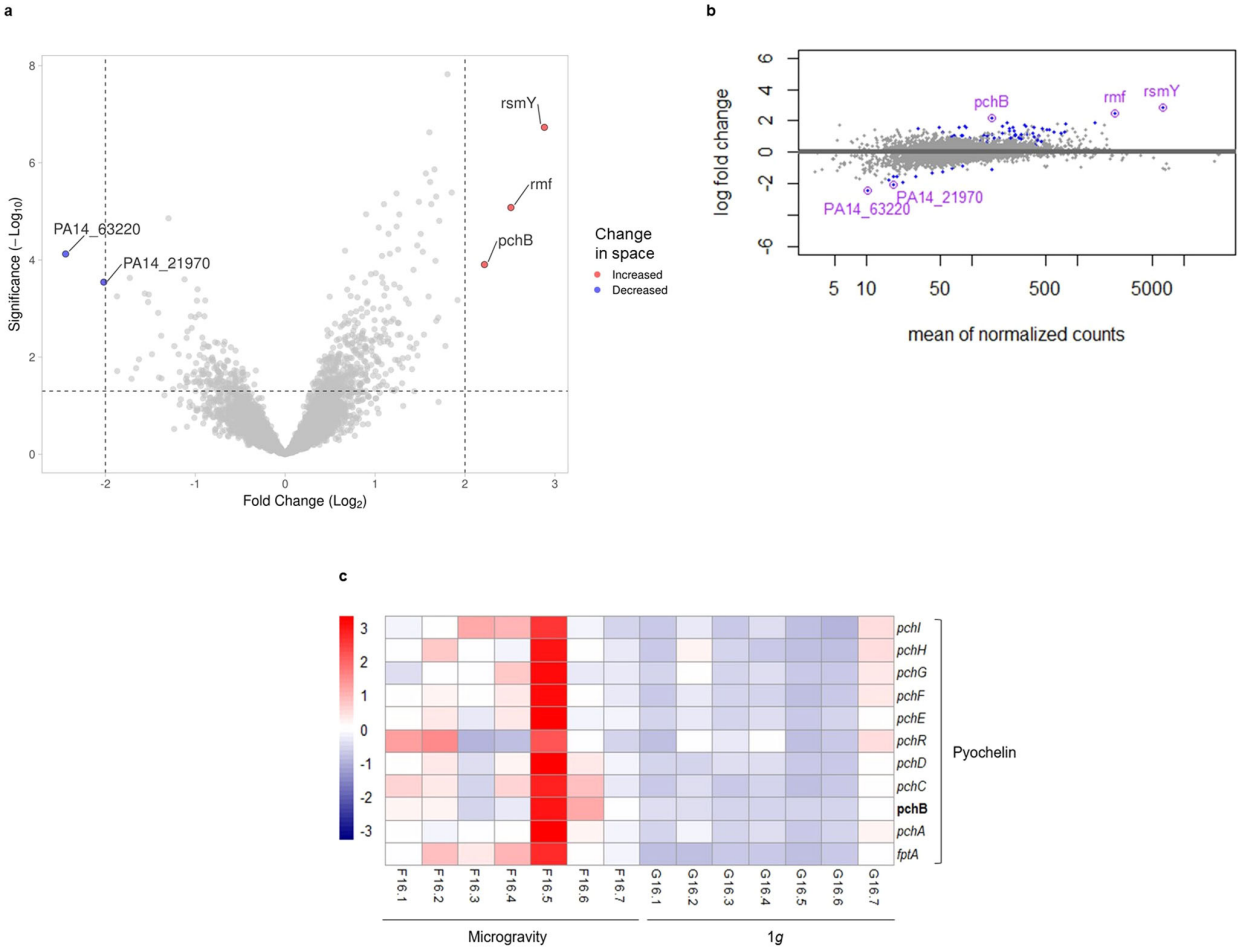
Transcriptional differences between *P. aeruginosa* biofilms formed on SS in space with respect to Earth. Transcriptomic data from 3-day-old biofilms grown on stainless steel coupons in microgravity with respect to 1 *g*. **a** Volcano plot and **b** MA plot of differential gene expression. **c** Heatmap of normalized counts for relevant genes involved in *P. aeruginosa* virulome, significant differentially expressed genes with fold change ≥2 in bold. Sample F16.5 was not considered an outlier because the expression of the rest of the genes was similar to the other samples and no problems were found in the quality control steps. *n* = 7 biological replicates per gravitational condition.

For SS, biofilms showed five DEG in microgravity with a fold change ≥2 (Fig. 6a, b, raw data in Supplementary Table 5). Three had increased expression (*rsmY* [PA0527.1], *rmf* [PA3049], *pchB* [PA4230]), and the remaining two had decreased expression in microgravity (PA14_63220 [PA4782], PA14_21970 [PA3249]). A KEGG pathways enrichment analysis was performed to understand at a bigger scale the more subtle changes (including the <2-fold changes) caused by microgravity (Supplementary Figure 6). No significant enrichments were found with >2-fold, but it showed the “nitrogen metabolism” pathway enriched (Supplementary Figure 7) in the space samples (1.02-fold). In addition, analyses were focused on genes involved in *P. aeruginosa* virulence and antimicrobial resistance. Despite *pchB* (2.2-fold) being the only gene in the virulome differentially expressed >2-fold, the other five genes involved in pyochelin production also had significant increased expression in microgravity for biofilms grown over SS (Fig. 6c): *pchI* (1.4-fold) [PA4222], *pchE* (1.9-fold) [PA4226], *pchD* (1.5-fold) [PA4228], *pchC* (1.9-fold) [PA4229], and *fptA* (1.8-fold) [PA4221]. There was no significant effect >2-fold in the resistome.

For LIS, biofilms in microgravity had only one DEG – PA14_08190 (−6.4-fold, raw data in Supplementary Table 6)—which codes for a hypothetical protein. This suggests that biofilms on Earth and in space formed over LIS have virtually the same transcriptome. Futures tests in microgravity should confirm if this holds true at the protein level and should consider studying the function of PA14_08190.

### Gene expression of biofilms on LIS vs biofilms on SS

The following paragraphs describe the results of the gene expression analyses performed to compare 3-day-old biofilms grown on LIS with respect to 3-day-old biofilms grown on SS (baseline). The first paragraph focuses on results under Earth’s gravity and the second paragraph for results in microgravity.

**Fig. 7:**
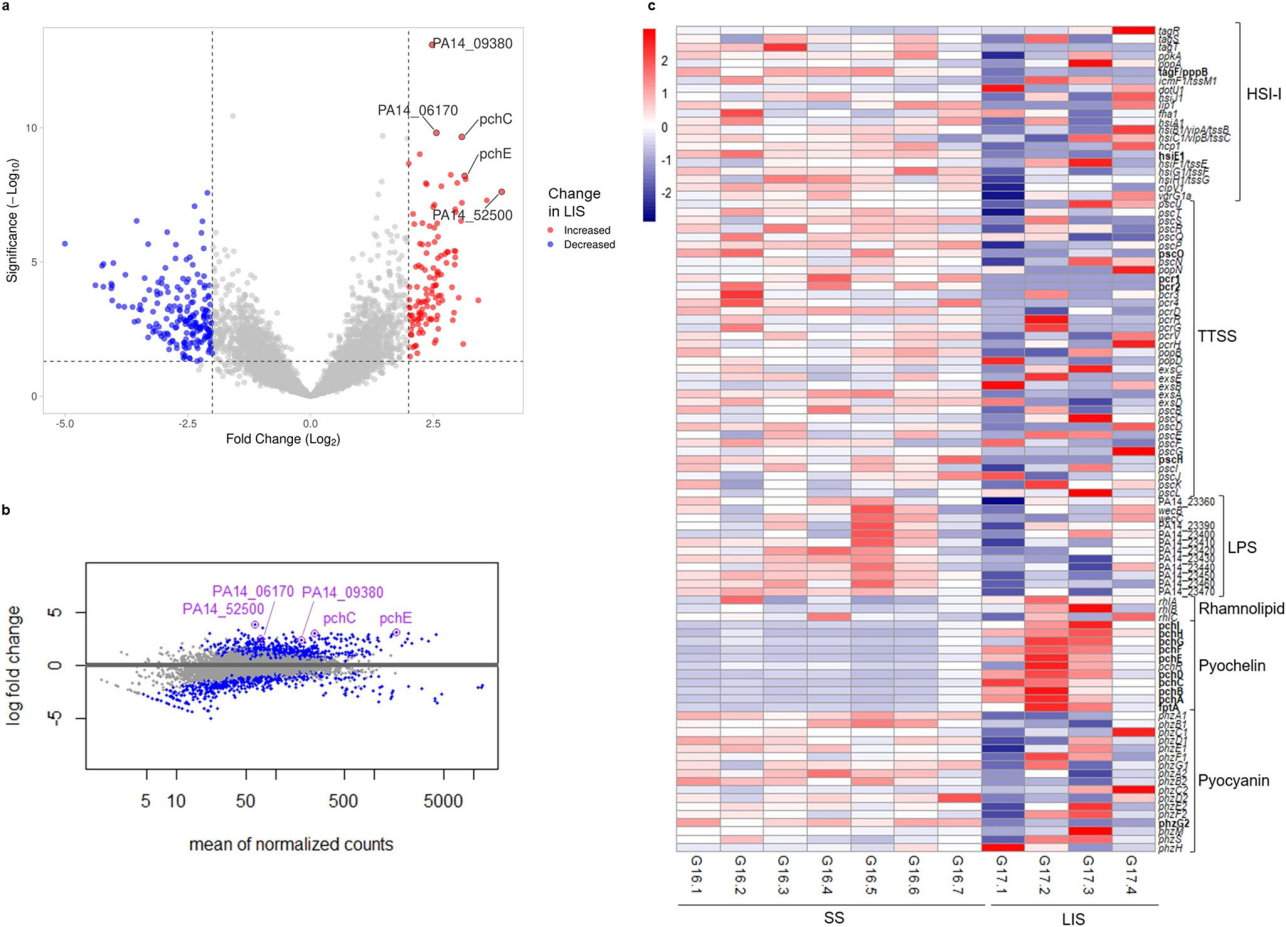
Transcriptional differences between *P. aeruginosa* biofilms formed on LIS with respect to SS, at 1 *g*. Transcriptomic data from 3-day-old biofilms. **a** Volcano plot and **b** MA plot of differential gene expression. **c** Heatmap of normalized counts for relevant genes involved in *P. aeruginosa* virulome, significant differentially expressed genes with fold change ≥2 in bold. For SS, *n* = 7 biological replicates. For LIS, *n* = 4 biological replicates.

On Earth, biofilms grown on LIS had 310 DEG with a fold change ≥2 (Fig. 7a, b, raw data in Supplementary Table 7) when compared to SS biofilms. The top five DEG were PA14_09380 [PA4218], *pchC*, PA14_06170 [PA1911], PA14_52500 [PA0909], and *pchE*; all with increased expression on LIS. Gene PA14_52500 is annotated as coding for a hypothetical protein and had the highest fold change (3.9) of all. Some of the genes that code for alginate production had significantly increased expression on LIS (below 2-fold): *mucB* (1.2-fold) [PA0764], *mucD* (1.1-fold) [PA0766], *algR* (1.5-fold) [PA5261], *mucE* (1.9-fold) [PA4033], *mucP* (1.4-fold) [PA3649]. The pathways enrichment analysis (Supplementary Figure 8) showed the “carbon metabolism” pathway as the most enriched on LIS (1.1-fold) (Supplementary Figure 9). On Earth, the virulome presented several genes of Hcp secretion island I (HSI-I), type three secretion systems (TTSS), lipopolysaccharide (LPS), and pyocyanin with decreased expression in LIS with respect to SS (Fig. 7c). Conversely, almost all the genes involved in synthesis of pyochelin had increased expression on LIS (>2.5-fold): *pchI* (3.1-fold), *pchH* (2.6-fold) [PA4223], *pchG* (2.7-fold) [PA4224], *pchF* (3.1-fold) [PA4225], *pchE* (3.1-fold), *pchD* (2.5-fold), *pchC* (3.1-fold), *pchB* (3.2-fold), *pchA* (3.0-fold) [PA4231], *fptA* (2.6-fold). In the resistome, gene *nouJ* [PA2645] had 2.2-fold increased expression on LIS. In addition, genes involved in apoptosis were differentially expressed on LIS biofilms, *rpoS* [PA3622] had increased expression (2.4-fold) and the antitoxins *higA* [PA4674] and PA14_21720 [PA2489] had decreased expression (−1.4 and −2.4-fold, respectively).

**Fig. 8:**
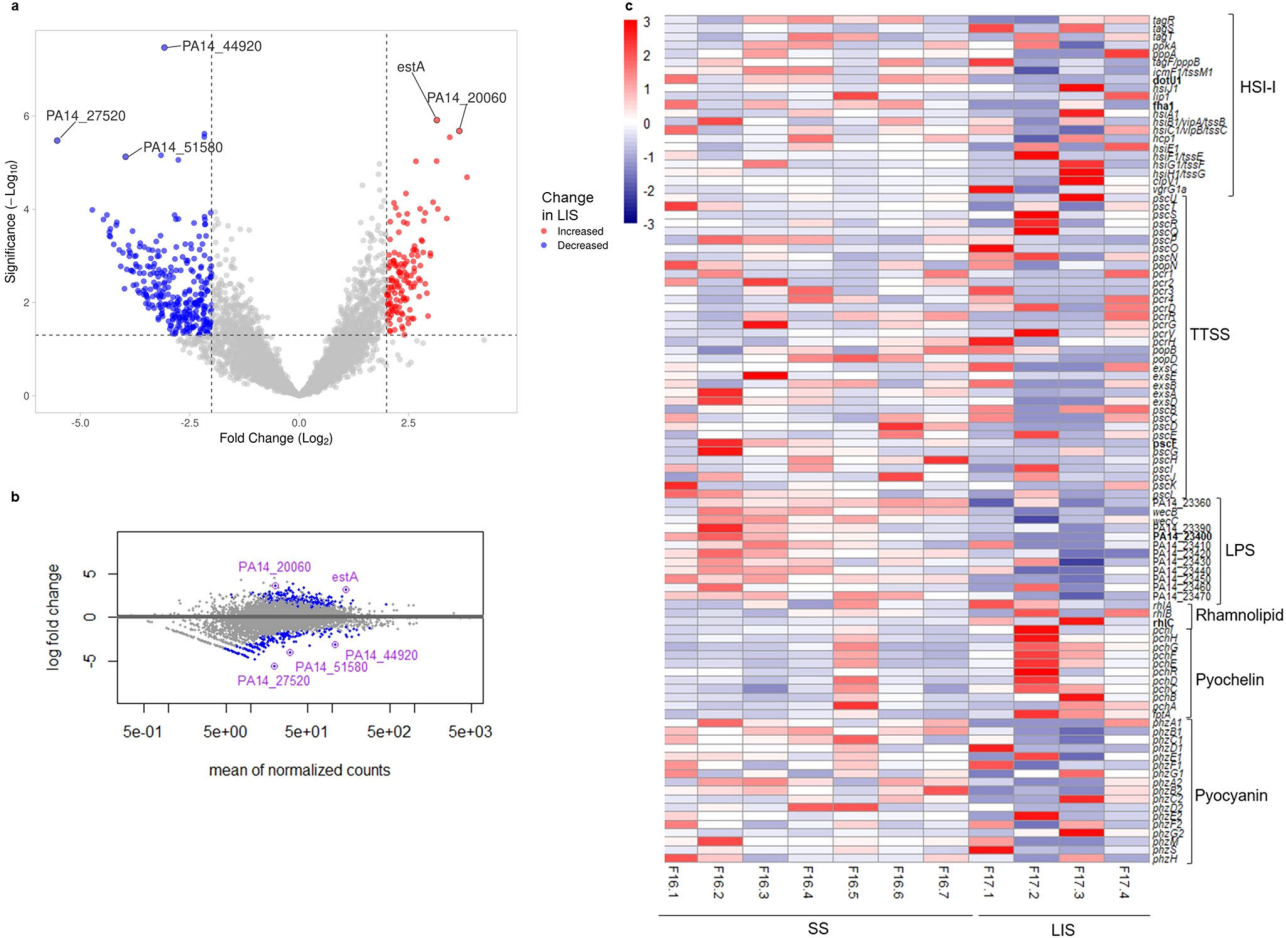
Transcriptional differences between *P. aeruginosa* biofilms formed on LIS with respect to SS, in microgravity. Transcriptomic data from 3-day-old biofilms. **a** Volcano plot and **b** MA plot of differential gene expression. **c** Heatmap of normalized counts for relevant genes involved in *P. aeruginosa* virulome, significant differentially expressed genes with fold change ≥2 in bold. For SS, *n* = 7 biological replicates. For LIS, *n* = 4 biological replicates.

In microgravity, biofilms grown on LIS had 425 DEG with a fold change >2 (Fig. 8a, b, raw data in Supplementary Table 8) when compared to SS. The top five DEG were PA14_27520 (−5.5-fold) [PA2826], PA14_44920 (−3.1-fold) [PA1509], PA14_20060 (3.7-fold), PA14_51580 (−4.0-fold), and estA (3.1-fold) [PA5112]. Three of these top five DEG code for hypothetical proteins that have not been characterized. In microgravity, no pathways were significantly enriched or depleted for LIS vs SS. Interestingly, the observations made on the virulome when comparing LIS with SS on Earth, remain true for biofilms on LIS in microgravity: HSI-I, TTSS, LPS, and pyocyanin-synthesis associated genes had reduced expression while genes associated with pyochelin and rhamnolipids had increased expression (Fig. 8c) with respect to SS. There was no increased expression of alginate-associated genes on LIS in microgravity, which differs to what was seen on Earth. In microgravity, the resistome of biofilms on LIS had increased expression of *mexT* (2.3-fold) [PA2492] and *galU* (2.1-fold) [PA2023], and decreased expression of *ampD* (−3.1) [PA4522]. Finally, when comparing LIS with respect to SS, no significant differences in gene expression of *rpoS* or *higA* were observed in microgravity, but one putative toxin (PA14_28120 [PA2781]) presented a 1.4-fold increased expression. Shared DEG in both gravitational regimes of biofilms on LIS with respect to SS at day 3 presented in Supplementary Table 9.

### Gene expression of 3-day-old biofilms vs 1-day-old biofilms

Changes in the transcriptome between 3- and 1-day-old biofilms provide insight into the biofilm formation process. The following paragraphs describe the results of the gene expression analyses performed to compare biofilms grown on SS for 3 days with respect to biofilms grown on SS for 1 day (baseline). The first paragraph focuses on results under Earth’s gravity and the second paragraph for results in microgravity.

On Earth, 3-day-old biofilms exhibited 115 DEG with fold change >2 (Supplementary Figure 10a, 10b, raw data in Supplementary Table 10) with respect to 1-day-old biofilms. The top five DEG at day three had decreased expression (PA14_40310 [PA1869], *trxB2* [PA0849], PA14_39700, PA14_10380 [PA4139], *nirN* [PA0509]). PA14_10380, the most differentially expressed (−5.2-fold), codes for a hypothetical protein. With respect to day one, day three had increased expression of several genes involved in production of Psl extracellular matrix polysaccharide, in particular *pslO* (3.1-fold) [PA2245]. The pathways enrichment analysis (Supplementary Figure 11) showed several depleted pathways at day three; “sulfur relay system” was the most depleted (−2.0-fold) (Supplementary Figure 12), followed by “nitrogen metabolism” (−1.7-fold) (Supplementary Figure 13). In the virulome of day three (Supplementary Figure 10c) there was a reduction in expression of genes in the Las (*lasA* −2.2-fold [PA1871], *lasB* −2.3-fold [PA3724]) and Rhl systems (*rhlA* −2.3-fold [PA3479], *rhlB* −2.2-fold [PA3478]), and of gene *algP/algR3* (−2.1-fold) involved in alginate synthesis. Regarding the resistome on Earth, day three presented decreased expression of genes that code for RND efflux pumps and outer membrane proteins: *mexH* (−2.1-fold) [PA4206], *ompA* (−2.6-fold) [PA3692], *opmD* (−2.3-fold) [PA4208].

In microgravity, 3-day-old biofilms showed 70 DEG with fold change ≥2 (Supplementary Figure S14a, S14b, raw data in Supplementary Table 11) with respect to 1-day-old biofilms. The top five DEG at day three correspond to: PA14_40310 (−2.6-fold), *malQ* (−3.1-fold) [PA2163], PA14_36650 (−3.7-fold) [PA2159], PA14_72370 (−5.1-fold) [PA5482] and *pchF* (3.2-fold). Again, the most differentially (under)expressed gene (PA14_72370) codes for a hypothetical protein. There was no systemic increased expression of extracellular matrix polysaccharides on day three in microgravity, but as on the ground, the gene *trxB2* (−2.3-fold) and the cytochrome c gene cluster (including *nirN* −2.1-fold) had decreased expression. The pathways enrichment analysis (Supplementary Figure 15) showed three depleted pathways at day three in microgravity; “Porphyrin and chlorophyll metabolism” is the most depleted (−2.0-fold) (Supplementary Figure 16). In the virulome (Supplementary Figure 14c), *rhlA* (−2.2-fold) was less expressed on day three (as seen on Earth) while all pyochelin-associated genes had increased expression (*pchI* 2.1-fold, *pchH* 2.4-fold, *pchG* 3.5-fold, *pchF* 3.2-fold, *pchE* 3.2-fold, *pchR* 1.3-fold [PA4227], *pchD* 2.6-fold, *pchC* 2.8-fold, *pchB* 3.3-fold, *pchA* 3.5-fold, *fptA* 2.5-fold). In the resistome, *ompA* (−2.4-fold) showed a reduced expression on day three in microgravity, as seen in the ground samples.

## Discussion

In this experiment, biofilm shape and general structure appearance was not affected by gravitational regime. Contrary to what has been previously reported for anaerobic *P. aeruginosa* PA14 biofilm growth in space^31^, we observed less robust biofilms in terms of biomass, thickness, and surface area coverage in microgravity with respect to 1 *g*. In microgravity, the increased expression of *rmf*, in charge of ribosomal hibernation^32^ during stationary phase^33^, could be indicative of a substrate-limited and byproduct-abundant extracellular environment for biofilms over SS, as the one theorized to occur in microgravity for non-motile planktonic cells^34,35^. Such an environment could be partially attributed to the increased planktonic cell growth in microgravity, increasing the accumulation of metabolic byproducts and depletion of nutrients, and potentially the cause of reduced biofilm formation. Proteomic analysis and functional analysis are needed to confirm these hypotheses.

Starvation environmental conditions can trigger biofilm dispersal in *P. aeruginosa*^36,37^. However, the genes known to be involved in biofilm dispersion^38^—*dipA* [PA5017]^39,40^, *rbdA* [PA0861]^41^, *nbdA* [PA3311]^42,43^, *gcbA* [PA4843]^44^, and *bdlA* [PA1423]^39^—were not differentially expressed in microgravity. Thus, biofilm dispersal could be regulated by other gene(s) in microgravity. Potentially, the increased planktonic growth and decreased biofilm formation are not a consequence of biofilm dispersion.

For SS, the most DEG in microgravity was *rsmY*. RsmY (small non-coding RNA) controls broad gene expression in *P. aeruginosa* through the sequestering of RsmA (gene repressor)^45^. RsmA targets the mRNAs of genes *mvaT* [PA4315], *pchB*, *pchC*, *pchD*, and *pchE*^46^ and, those genes demonstrated increased expression in microgravity. We hypothesize that RsmY could be regulating some of the biggest transcriptional changes observed in biofilms grown over SS in microgravity. It is worth nothing that DEG PA14_63220 and PA14_21970 code for a hypothetical protein and a transcriptional regulator, respectively, that have not yet been characterized and whose functions could shed light to the changes observed in microgravity. Moreover, this could be specific for biofilms growing on SS, only for biofilms past microcolony stages, or both, as biofilms on LIS did not have increased expression of *rsmY* in microgravity.

Our results suggest potential increased virulence of PA14 3-day-old biofilms on SS in microgravity through increased expression of pyochelin. Pyochelin is secreted to chelate iron for bacterial use^47^ and the outer membrane receptor, FptA, internalizes it. In addition, pyochelin production in biofilms is characteristic of aggressive and chronic infections^48,49^. Proteomic tests should confirm these results at protein level and on different surfaces.

The green, brown, and pink colors of the liquid culture in microgravity could be attributed to the production of pigmented virulence factors such as pyoverdine (green-brown to green-yellow), pyorubin (red), pyocyanin (deep blue), pyomelanin (black)^50^. It appears that *P. aeruginosa* had an increased production of pyoverdine in the first days which then transitioned to an increased production of pyorubin. Interestingly biofilms in microgravity had no increased expression of pyoverdine, so the green-brown color in the liquid culture could be attributed to a higher production of this virulence factor by the planktonic cells. On the other hand, the pink color, possibly reflecting the production of pyorubin, could not be assessed as the genes involved in pyorubin production have not been identified in PA14, to our knowledge. These hypotheses need to be confirmed with transcriptomics and proteomics analyses of the planktonic cultures in future experiments. Nevertheless, the potential increased production of pigmented virulence factors of *P. aeruginosa* PA14 planktonic cells in microgravity, when grown in nutrient-rich medium, may pose a health risk for infections in space.

Remarkably, LIS’s biofilm inhibition on Earth, was even greater in microgravity. Not only were biomass and surface area minimized, but these parameters stayed almost constant in time for microgravity biofilms on LIS. Coupled with the significant biofilm thickness reduction in time in microgravity, contrary to the increasing thickness trend on Earth, LIS performance in space makes it suitable to reduce biofilm formation in spacecraft components. Previous studies with different LISs show that this type of surfaces greatly reduce the attachment of both gram-positive and gram-negative bacteria (including *P. aeruginosa*)^25,51–55^. The liquid-like properties of LISs are believed to be the essential for the bacterial attachment inhibition^56^. Interestingly, biofilm formation on LIS was observed only in areas without the nucleic acid layer. We hypothesize that the thin layer of nucleic acids (DNA, RNA, or both) contributes to attachment inhibition.

Nucleic acids have a hydrophilic phosphate backbone (negatively charged), and hydrophobic nitrogen bases^57–59^. It is possible that the interface between liquid culture and oil layer provided an ideal zone for DNA/RNA interaction, leading to their immobilization on the surface. It is well-established that oil/water interfaces can capture protein and nucleic acids^60^; and in some cases, the surface forces are strong enough to denature proteins^60–67^. Characterization of the protein adsorption/denaturing process on oil-infused textured surfaces, as LIS, remains to be done^68,69^. Thus, we hypothesize that the oil/water interface forces could be enough to destabilize extracellular DNA (eDNA), inducing conformational changes that result in its unzipping to ssDNA^70^, and that the DNA-associated proteins can be denatured to expose the DNA charges. Such that the hydrophobic portions of DNA/RNA partition into the oil of the LIS, leaving the hydrophilic phosphate backbone facing toward the aqueous culture. The mechanism of nucleic acid adsorption onto LIS surface and the exact composition of this layer remains to be elucidated and more tests are needed to confirm this hypothesis.

**Fig. 9:**
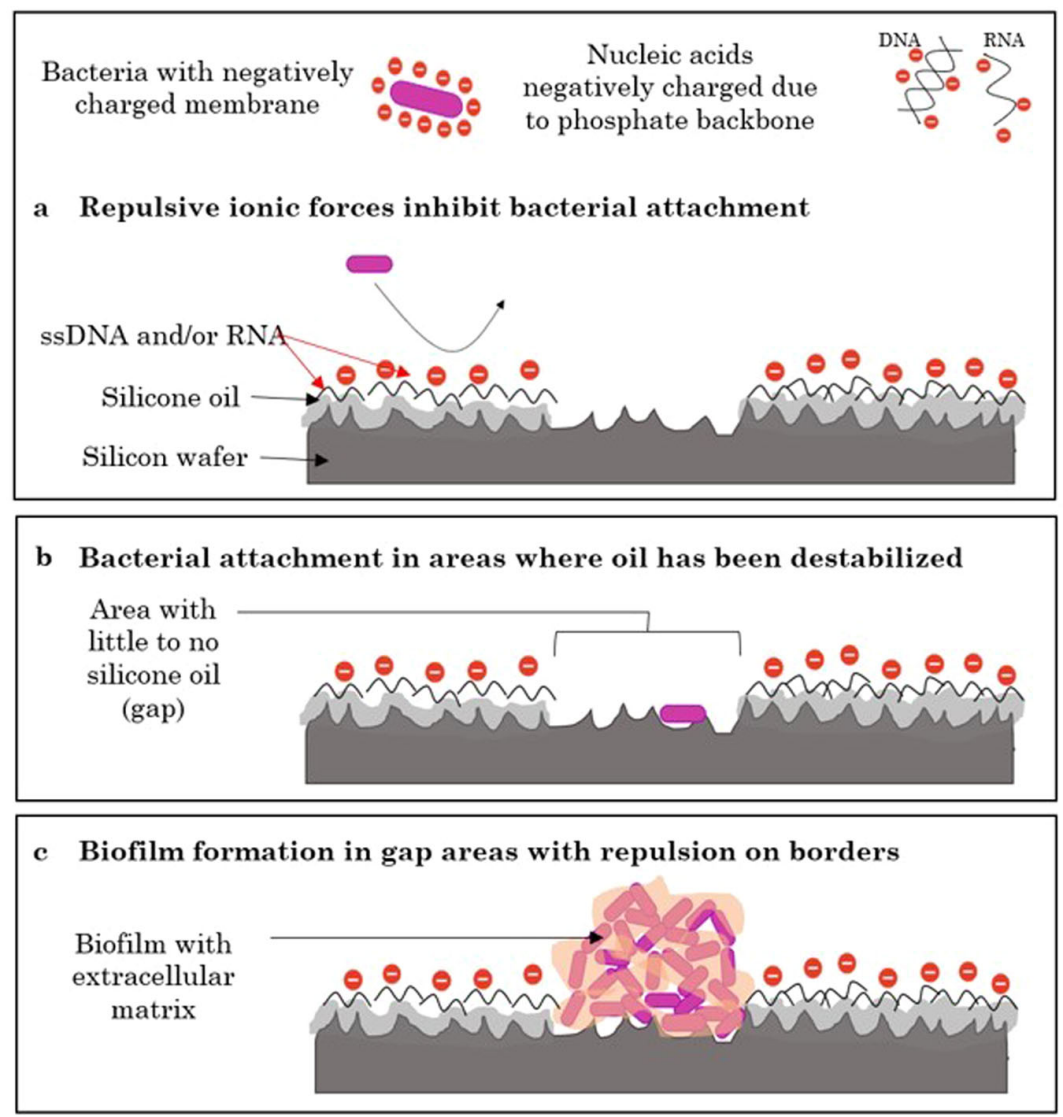
Proposed mechanism contributing to biofilm inhibition on LIS surface. Both bacteria and nucleic acids possess a negative charge. **a** When the nucleic acids get immobilized on the LIS surface, this creates a layer of negatively charged molecules that can repel bacteria through ionic forces. **b** Bacteria can attach to areas of the LIS where there is a gap in the oil coat, previously destabilized. **c** Biofilms form in these gap areas but grow with sharp edges due to the repulsive forces on the borders.

*Pseudomonas aeruginosa* (negatively charged due to its LPS) could be repelled by the phosphates of the nucleic acid layer (Fig. 9). Recent studies show reduced attachment of *P. aeruginosa* on surfaces with covalently attached and adsorbed DNA^71^, and on surfaces with DNA-poly(ethyleneimine) multilayers^72^. *Staphylococcus* was also repelled by multilayer chitosan-DNA coatings^73^. This hypothesis implies that DNA/RNA was released by the planktonic cells and attached to LIS early in the experiment. On LIS, biofilm’s reduced expression of LPS-related genes (on Earth and in microgravity) and increased expression of alginate (only on Earth)—a secreted polyanionic polysaccharide that helps mask LPS’s charge—could be related to the presence of nucleic acid repulsion forces.

In solid abiotic surfaces, the deposition of eDNA is required for initial microcolony formation in *P. aeruginosa* biofilms^74–77^. The eDNA is located in the active migration zone of the biofilm and plays a role in its expansion^78^. Thus, the nucleic acid layer on LIS could represent a risk of delayed/increased biofilm formation for long term. However, it is possible that the orientation and immobilization of DNA/RNA plays a role in adhesion inhibition. In addition, the physics of bacterial interactions at interfaces (specially oil/water interfaces) is unclear and can differ from usual biofilm formation^79^. More tests with longer incubation periods are needed to determine the time limit (if any) of the observed biofilm inhibition on LIS.

Although LIS is typically stable, it is possible that protein/RNA acted as a surfactant and led to destabilization of the oil^80^. The biofilm increased expression of *rhlC* in microgravity could have resulted in increased rhamnolipid (biosurfactants)^81^ production, contributing to such destabilization. Interestingly, *P. aeruginosa* planktonic cells in microgravity have been reported to produce larger amounts of rhamnolipids and this could be the case for biofilms on LIS as well^82^.

The gene expression analysis of biofilms formed on LIS with respect to SS suggests several other hypotheses of mechanisms contributing to the reduced biofilm formation: (1) Increased expression of pyochelin genes (on Earth and in microgravity) and of rhamnolipid genes (only on Earth), which are produced in nitrogen-limited environments^83^, could reflect exacerbated nutrient depletion in the extracellular environment of LIS biofilms in comparison with SS. It is possible that the lubricant oil could be self-spreading over the biofilms^19^ and thereby limiting nutrient transport. (2) Increased expression of rhamnolipid genes, including *estA*, an esterase that promotes rhamnolipid production^84^. Rhamnolipids sometimes lead to biofilm detachment^83^. (3) Increased apoptosis as observed in previous spaceflight studies^34^. Apoptosis of biofilms on LIS could be mediated by the increased expression of *rpoS* on Earth (global stress regulator), underexpression of antitoxins on Earth (*higA*, PA14_21720) or increased expression of toxins in microgravity (PA14_28120) with respect to SS. We acknowledge that transcriptome changes are not direct evidence of protein levels nor cell functions, and that more tests are needed to confirm these hypotheses.

It is important to consider that growing biofilms over LIS could affect virulence and antimicrobial resistance. However, predicting such an effect is complicated as there is gene expression evidence for increasing and decreasing effects compared to SS. On one side, biofilms on LIS had increased expression of the virulence factors pyochelin and rhamnolipids. On the other side, biofilms on LIS presented a general decreased expression of type 6 secretion systems from the HSI-I loci, which could make them less capable of establishing chronic infections^85^.

LIS seems to prevent the first stage of biofilm formation: cell attachment. Nevertheless, more tests and analysis are needed to fully understand the mechanisms behind this phenomenon. In parallel, elucidating the functions of the uncharacterized genes that were differentially expressed on LIS with respect to SS can provide further insight. Based on the minimized biofilm formation on LIS in microgravity, it presents itself as a good option for use in spacecraft components. In addition, the use of directly immobilized DNA/RNA over surfaces (e.g., using covalent bonding, physical adsorption, or streptavidin-biotin immobilization^86,87^) should also be explored to reduce bacterial attachment and biofilm formation in susceptible spacecraft components.

Biofilms on LIS in microgravity seemed to remain in the microcolony stage for days 2 and 3, which could explain the limited differential gene expression between 1-day-old biofilms and 3-day-old biofilms. 3-day-old SS biofilm gene expression profile suggests a potential decrease in metabolic activity (reduced nitrogen metabolism) and quorum sensing signaling (reduced expression of Las and Rhl systems) with respect to 1-day-old biofilms. Further tests are needed to confirm such hypothesis but it agrees with SS biofilm morphology, as expression of these systems decreases as biofilm height increases^88^.

Bacterial growth dynamics can differ in space with respect to behavior observed on Earth^89^. Hence, it must be noted that comparing bacterial cultures and biofilms from a given moment in time (e.g., “day 3”) may be doing so at different stages of growth, e.g., in a gravitational condition it may still be at the end of exponential phase while at another it may already be stationary. Acknowledging the limitation of getting data from three instantaneous moments in time in our experiment, we recommend that future studies (i) acquire continuous data as possible, and (ii) also characterize the growth stage and gene expression of the planktonic cell culture. Staining protocols may be improved with the use of a third dye to target polysaccharides. Furthermore, future studies may interrogate our observations further by determining the precise composition of the nucleic acid layer observed on LIS. Future studies, namely those focused on spacecraft water processing assemblies may improve on the fidelity of their study by using Ersatz Wastewater formulation to replicate the wastewater as received on the ISS’ system, extend the duration of the experiment to multiples of months as applicable, and the substrate material may be changed to that of the specific component being studied.

## Methods

### Reagents and hardware

The reagents and hardware were prepared as described in Flores et al. (2022)^18^. In brief, the PBS was prepared with PBS pellets (Sigma-Aldrich, Cat. P4417) and autoclaved. The 1X LBK medium was LB Lennox (Sigma, Cat. L3022) supplemented with 0.86% potassium nitrate (Sigma, Cat. P8291) and filter sterilized with 0.22 µm filters (Nalgene, Cat. 566-0020). LBK medium was prepared at 1.18X concentration to load in the hardware to account for dilution during experiment activation. The 11.4% PFA solution was prepared by diluting 16% PFA (Alfa Aesar, Cat. 43368) with PBS. When mixed with the sample it yields a final concentration of 4% PFA. The RNAlater (Invitrogen, Cat. AM7021) was refrigerated prior to loading it in the hardware to induce the formation of RNAlater crystals. These crystals were then pulverized and the RNAlater was loaded cold into the hardware. Preemptive formation and pulverization of the RNAlater crystals avoided the formation of long crystals during launch (4 °C) that could cause sample loss, early activation, and leaks by displacing the hardware chambers. After sample incubation, the RNAlater was still efficient at preserving RNA from biofilms^18^.

BioServe’s FPA housed in the Group Activation Pack (GAP) were used as both ground and flight sample hardware. The FPA hardware components were cleaned with 1% Liquinox (Alconox, Cat. 1201-1), coated with sigmacote (Sigma-Aldrich, Cat. SL2), and dried at 100 °C for 30 min to siliconize the glass and facilitate sample assembly.

### Bacterial strain and Inoculum

All the biofilm experiments were performed using *Pseudomonas aeruginosa* UCBPP-PA14 strain, which was kindly provided by Dr. George O’Toole. This strain was selected for the project to replicate the column-and-canopy biofilm structure previously observed in microgravity by Kim et al. (2013)^31^ when growing PA14 biofilms over cellulose membrane. The results of the biofilms grown over cellulose membrane are reported in a separate publication^20^.

The bacterial inoculum was prepared with 6 ml of LBK media inoculated with PA14 and incubated at 37 °C for 16 h (final OD_595_ 0.706). The overnight inoculum was diluted 1:100 with PBS (final OD_595_ 0.001) to maintain cells in stasis. The diluted inoculum was used to load the second chamber of FPAs.

### Test surface preparation

Biofilms were grown on 1 cm^2^ material coupons of SS316 (MPPR-311-6), pSS316, and LIS. SS316 coupons were cleaned with 1% Liquinox (Alconox Cat. 1201-1). For the pSS316, clean SS316 coupons were passivated with citric acid (Sigma-Aldrich, Cat. C-8385). Preparation of LIS coupons (silicon wafer impregnated with silicone oil) has been described in detail previously^1,18,19^. LIS coupons were not cleaned, to avoid removing the silicone oil, but they were prepared in sterile conditions. LIS’s silicone oil layer (0.5–1.5 µm in thickness) has no bactericidal nor growth boost effect on *P. aeruginosa* PA14^18^.

### Sample preparation

A total of 144 samples were prepared, 72 for flight and 72 for corresponding ground controls. All coupons were prepared in the same fashion. Each gravitational condition tested three material surfaces (SS316, pSS316, and LIS), three biofilm incubation times (1, 2, or 3 days), and two termination reagents (PFA for morphology analysis and RNAlater for transcriptomics) with four replicates per condition. Flight samples were sent to the ISS on the Cygnus NG-12 spacecraft.

Double-sided tape (3 M, Cat. 9731) was placed on the backside of the coupons to adhere them onto the inserts. Adhered coupons on inserts were installed in the FPAs and autoclaved. FPAs were loaded with 2.75 ml of sterile 1.18X LBK and the first chamber was closed with a second insert. The use of solid inserts prevents gas exchange and forces anaerobic growth of *P. aeruginosa*. The first chamber was incubated at 37 °C for 24 h as a contamination check. Afterward, the second chamber was loaded with 0.5 ml of the PA14 inoculum in stasis and closed with a rubber septum. The third and last chamber was filled with 1.75 ml of PFA or RNAlater and closed with a rubber septum. Eight FPAs with the three-chamber configuration were assembled into each GAP and transferred to 4 °C.

### Biofilm formation assays

Spaceflight samples were launched in stasis (in PBS and at 4 °C) to the ISS to avoid bacterial growth before microgravity conditions were achieved. Once in microgravity, astronauts Jessica Meir and Christina Koch activated the spaceflight samples by combining the first and second chambers of the FPAs, which introduced the inoculum into the culture media. Samples were incubated at 37 °C inside BioServe’s Space Automated Bioproduct Lab (SABL) for the designated incubation times (1, 2, or 3 days) in anaerobic conditions. At the end of incubation, the third chamber was introduced to fix (PFA) or preserve (RNAlater) the samples in microgravity, and samples were transferred to 4 °C or −80 °C, respectively, until unberthing. Fixed and preserved samples then returned to Earth at 4 °C and −20 °C, respectively. Once on Earth, samples were stored at 4 °C (fixed) and −80 °C (preserved) until sample processing.

Ground samples were performed asynchronously, activating and terminating two hours later to replicate the order of flight procedures. The temperature profile of spaceflight samples was also reproduced for the ground samples. When spaceflight samples returned to Earth, all coupons (both flight and ground) were recovered from the FPAs and processed for morphology analysis (PFA) or transcriptomic analysis (RNAlater).

### Planktonic cell analysis

Despite biofilms being the focus of our experiment, the planktonic cell culture of the samples was visually inspected with the naked eye for notorious changes between samples. In addition, the liquid cultures’ optical density at 600 nm (OD_600_) was measured. Samples were vortexed prior to measuring the OD_600_ to avoid cell clumping. Due to spaceflight constraints and the interest of fixing the samples while in microgravity, it was not possible to measure CFU/mL at experiment’s end. However, for *P. aeruginosa* PA14 in our experimental configuration, the OD_600_ measurements allow correct distinction of the bacterial stages of growth and are correlated to the viable cell density (Supplementary Figures 3 and 4).

### Biofilm morphology analysis

To eliminate the planktonic cells, the coupons fixed in PFA were washed three times with 1 mL of PBS which was slowly poured on the walls of the wells to avoid disturbing the biofilm. The samples were stained with PI [15 µM] (PromoKine Cat. PK-CA707-40017 diluted with water) for 15 min and with FilmTracer™ FM™ 1-43 Green Biofilm [10 µg/ml] (Invitrogen Cat. F10317 diluted with DMSO and water as per manufacturer’s instructions) for 30 min under light-protected conditions. After each stain the samples were washed with 1 ml of PBS. Immediately after the wash, the PBS was removed and the stained samples were mounted onto glass slides; gorilla glue was used to adhere the coupon to the slide and the biofilm on top was covered with one drop of VectaShield HardSet™ Antifade Mounting Medium (Vector Laboratories, Cat. H-1400-10) and a coverslip. Mounted samples were stored at 4 °C for 20 h protected from light prior to imaging, this allowed the mounting medium and glue to harden in order to avoid smearing the biofilm while imaging. Samples were imaged in groups of 4 per session (spaceflight samples with their corresponding ground controls in the same session).

The Nikon SIM/A1 Laser scanning Confocal Microscope (inverted) of CU Boulder Light Microcopy Core Facility was used to take four 125 × 125 µm Z-stacks (30–40 slices with step size ranging from 0.1–1.5 µm, average 0.5 µm) per coupon, aligning the four fields of view to the center of the coupon. The biofilms were imaged with 100X SR Apo immersion oil objective (NA = 1.49 WD = 120 µm), using lasers 488 nm (green, emission filter 500–550 nm) and 561 nm (red, emission filter 575–625 nm). All images were acquired with 1.2 pinhole, laser power range (green and red = 0.1 to 2.3), offset range (green = −48 to 4, and red = −45 to 6), and PMT HV range (green = 3.0 to 86, red = 2.0 to 82) using the NIS Elements software. Images were taken with laser powers that avoided photobleaching and HV sensitivity settings that maximized the dynamic range without saturation. Resulting biofilm images were pseudo-colored with red (PI, nucleic acids) and green (Green Biofilm, lipids) for display with a linear LUT covering the full range of the data. Raw image files were used for quantitative analysis. First, Image J was used to merge both fluorescent channels and convert files from nd2 to tiff format. Then, the “Slice Remover” plugin was used to eliminate the slices with no biofilm signal from each Z-stack. The “Convert to OME-Tiff” macro was used to generate compatible files for analysis with the publicly available COMSTAT2 software^90–92^. COMSTAT2 calculated biofilm mass (µm^3^/µm^2^), thickness (µm), and surface area coverage (%) of the biofilms with automatic thresholding.

### Biofilm transcriptomic analyses

Samples preserved in RNAlater were thawed at room temperature and washed 3 times with 1 ml PBS to get rid of any planktonic cells. PBS was removed and 1 ml of fresh RNAlater was added. The samples were ultrasonicated for 15 min at 40 kHz, followed by 5 min of vortexing to detach biofilm bacterial cells from the surface materials. RNA extraction was performed by University of Colorado Boulder BioFrontiers Sequencing Core and the sequencing by the Anschutz Genomics Shared Resource. The RNAlater with the suspended biofilm cells was centrifuged, and the pellet was used to extract total RNA using the Quick-RNA Fungal/Bacterial microprep Kit (Zymo Research Cat. R2010). RIN values were measured for the RNA extracted (Supplementary Table 12) RNA sequencing libraries were prepared using SMARTer Stranded Total RNA-Seq Kit v3 (Takara Cat. 634485) and paired-end sequencing was performed on Illumina NOVASeq 6000 with 40 million reads per sample (Additional Transcriptomic information presented in Supplementary Table 13).

Transcriptomic analysis was performed on CU Boulder’s RMACC Summit supercomputing cluster. Raw sequencing files were checked for quality control using FastQC (version 0.11.9)^93^ and multiQC (version 1.0.dev0)^94^. Then Illumina adapter contamination was eliminated using Trimgalore (version 0.6.6) with cutadapt (version 2.6)^95^, after which quality control was performed again. Trimmed reads were mapped to PA14 rRNA sequences to remove the contamination, the resulting unmapped reads were then mapped to the Ensembl Bacteria reference genome of *Pseudomonas aeruginosa* UCBPP-PA14 (accession number GCA_000014625), both mapping steps were performed using Bowtie2 (version 2.4.4)^96^. The mapping results were sorted, indexed, and converted from .sam files to .bam files using SAMtools (version 1.11)^97^. The reads per gene were counted with Feature Counts (Rsubread package version 2.8.2)^98^, and differential gene expression analyses were done in an R (version 4.1.1)^99^ with DESeq2 (version 1.34.0)^100^. Pathways enrichment analyses were performed online using ESKAPE Act PLUS^101^ and the whole list of DEG (including ≤2-fold change genes) per condition as input.

### Statistics

Morphology data was tested for parametric assumption compliance using Shapiro Wilk and Levene’s tests. The data did not comply with all the assumptions, thus non-parametric tests were used for all statistical analyses. Biofilm biomass, thickness, and surface area coverage data was compared between groups using two-sided Kruskal Wallis and Dunn’s (with Bonferroni correction) tests to determine if differences observed were significant. Significant differences have the significance level specified in parenthesis or in the plot (minimum of *p*-value < 0.05), while the non-significant values correspond to *p*-value > 0.05. Differential gene expression analysis was performed with DESeq2 using *p*-value < 0.05 as significance threshold, except for comparison between SS316 and pSS316 which used *p*-adj ≤ 0.05. Pathways enrichment analysis was performed with ESKAPE Act PLUS using a binomial test (FDR-corrected) with *α* = 0.05.

### Reporting summary

Further information on research design is available in the Nature Research Reporting Summary linked to this article.

### Supplementary information

Supplemental Information

Supplementary Table 1

Supplementary Table 2

Supplementary Table 3

Supplementary Table 4

Supplementary Table 5

Supplementary Table 6

Supplementary Table 7

Supplementary Table 8

Supplementary Table 9

Supplementary Table 10

Supplementary Table 11

Supplementary Table 13

Reporting Summary

## Data Availability

Data available on NASA’s Open Science Data Repository (OSDR, https://osdr.nasa.gov/bio/). The microscopy data under study OSD-627 and 10.26030/bp7m-0f62^102^. The transcriptomic data under study OSD-554 10.26030/d5dg-7s68^103^. The data will also be available on NASA’s Physical Sciences Informatics (PSI) data repository (https://psi.nasa.gov).

## Code availability

The pipeline code used for the transcriptomic analysis is available on NASA’s Open Science Data Repository (OSDR, https://osdr.nasa.gov/bio/) under study OSD-554 10.26030/d5dg-7s68^103^.

## References

[1] Zea L (2018). Design of a spaceflight biofilm experiment. Acta Astronaut..

[2] Zea L (2020). Potential biofilm control strategies for extended spaceflight missions. Biofilm.

[3] Weir, N. et al. Microbiological characterization of the international space station water processor assembly external filter assembly S/N 01. in *42nd International Conference on Environmental Systems* (American Institute of Aeronautics and Astronautics, 2012).

[4] Carter, D. L. et al. Status of ISS water management and recovery (2017-036). in *47th International Conference on Environmental Systems* (2017).

[5] Klintworth R, Reher HJ, Viktorov AN, Bohle D (1999). Biological induced corrosion of materials II: new test methods and experiences from mir station. Acta Astronaut.

[6] Mansour R, Elshafei A (2016). Role of microorganisms in corrosion induction and prevention. Br. Biotechnol. J..

[7] Kaksonen AH (2021). Potential of Acidithiobacillus ferrooxidans to grow on and bioleach metals from mars and lunar regolith simulants under simulated microgravity conditions. Microorganisms.

[8] Santomartino R, Zea L, Cockell CS (2022). The smallest space miners: principles of space biomining. Extremophiles.

[9] Kaksonen AH (2020). Prospective directions for biohydrometallurgy. Hydrometallurgy.

[10] Pyle, B. H. et al. Bacterial Growth on surfaces and in suspensions. European Space Agency, Biorack on Spacehab. SP-1222 (1999).

[11] Hall-Stoodley L, Costerton JW, Stoodley P (2004). Bacterial biofilms: from the Natural environment to infectious diseases. Nat. Rev. Microbiol..

[12] Seil JT, Webster TJ (2012). Antimicrobial applications of nanotechnology: methods and literature. Int. J. Nanomed..

[13] Santos, C., Albuquerque, A., Sampaio, F. & Keyson, D. Nanomaterials with antimicrobial properties: applications in health sciences. in *Microbial Pathogens and Strategies for Combating Them: Science, Technology and Education* (Formatex Research Center, 2013).

[14] Puckett SD, Taylor E, Raimondo T, Webster TJ (2010). The relationship between the nanostructure of titanium surfaces and bacterial attachment. Biomaterials.

[15] Oh YJ, Lee NR, Jo W, Jung WK, Lim JS (2009). Effects of substrates on biofilm formation observed by atomic force microscopy. Ultramicroscopy.

[16] Lorite GS (2013). Surface physicochemical properties at the micro and nano length scales: role on bacterial adhesion and Xylella fastidiosa biofilm development. PLoS ONE.

[17] Walther BA, Ewald PW (2004). Pathogen survival in the external environment and the evolution of virulence. Biol. Rev. Camb. Philos. Soc..

[18] Flores, P. et al. Preparation for and performance of a *Pseudomonas aeruginosa* biofilm experiment on board the International Space Station. *Acta Astronaut*. 10.1016/j.actaastro.2022.07.015 (2022).

[19] Smith JD (2013). Droplet mobility on lubricant-impregnated surfaces. Soft Matter.

[20] Flores, P., Luo, J., Mueller, D. W., Muecklich, F. & Zea, L. *Space Biofilms—An Overview of the Morphology of Pseudomonas aeruginosa Biofilms Grown on Silicone and Cellulose Membranes on Board the International Space Station* (International Astronautical Federation, 2022). 10.1016/j.bioflm.2024.100182 PMC10869243 38370151

[21] San Marchi C, Somerday BP, Tang X, Schiroky GH (2008). Effects of alloy composition and strain hardening on tensile fracture of hydrogen-precharged type 316 stainless steels. Int. J. Hydrog. Energy.

[22] Maller RR (1998). Passivation of stainless steel. Trends Food Sci. Technol..

[23] Lafuma A, Quéré D (2011). Slippery pre-suffused surfaces. EPL.

[24] Ban G-H, Lee J, Choi C-H, Li Y, Jun S (2017). Nano-patterned aluminum surface with oil-impregnation for improved antibacterial performance. LWT.

[25] Epstein AK, Wong T-S, Belisle RA, Boggs EM, Aizenberg J (2012). Liquid-infused structured surfaces with exceptional anti-biofouling performance. PNAS.

[26] Subramanyam SB, Azimi G, Varanasi KK (2014). Designing lubricant-impregnated textured surfaces to resist scale formation. Adv. Mater. Interfaces.

[27] Hardt S, McHale G (2022). Flow and drop transport along liquid-infused surfaces. Annu. Rev. Fluid Mech..

[28] Hauer L (2021). How frost forms and grows on lubricated micro- and nanostructured surfaces. ACS Nano.

[29] McBride SA, Dash S, Varanasi KK (2018). Evaporative crystallization in drops on superhydrophobic and liquid-impregnated surfaces. Langmuir.

[30] Xiao L (2013). Slippery liquid-infused porous surfaces showing marine antibiofouling properties. ACS Appl. Mater. Interfaces.

[31] Kim W (2013). Spaceflight promotes biofilm formation by *Pseudomonas aeruginosa*. PLoS ONE.

[32] Maki Y, Yoshida H (2021). Ribosomal hibernation-associated factors in *Escherichia coli*. Microorganisms.

[33] Jaishankar J, Srivastava P (2017). Molecular basis of stationary phase survival and applications. Front. Microbiol..

[34] Zea, L. *Phenotypic and Gene Expression Responses of E. coli to antibiotics during Spaceflight* (University of Colorado Boulder, 2015).

[35] Zea L (2016). A Molecular genetic basis explaining altered bacterial behavior in space. PLoS ONE.

[36] Gjermansen M, Ragas P, Sternberg C, Molin S, Tolker-Nielsen T (2005). Characterization of starvation-induced dispersion in Pseudomonas putida biofilms. Environ. Microbiol..

[37] Ott E (2019). Molecular response of Deinococcus radiodurans to simulated microgravity explored by proteometabolomic approach. Sci. Rep..

[38] Valentini M, Filloux A (2016). Biofilms and cyclic di-GMP (c-di-GMP) signaling: lessons from *Pseudomonas aeruginosa* and other bacteria. J. Biol. Chem..

[39] Li Y (2014). BdlA, DipA and induced dispersion contribute to acute virulence and chronic persistence of *Pseudomonas aeruginosa*. PLOS Pathog..

[40] Roy AB, Petrova OE, Sauer K (2012). The phosphodiesterase DipA (PA5017) is essential for *Pseudomonas aeruginosa* biofilm dispersion. J. Bacteriol..

[41] An S, Wu J, Zhang L-H (2010). Modulation of *Pseudomonas aeruginosa* biofilm dispersal by a cyclic-Di-GMP phosphodiesterase with a putative hypoxia-sensing domain. Appl. Environ. Microbiol..

[42] Li Y, Heine S, Entian M, Sauer K, Frankenberg-Dinkel N (2013). NO-induced biofilm dispersion in *Pseudomonas aeruginosa* is mediated by an MHYT domain-coupled phosphodiesterase. J. Bacteriol..

[43] Basu Roy, A. & Sauer, K. Diguanylate cyclase NicD-based signalling mechanism of nutrient-induced dispersion by *Pseudomonas aeruginosa*. *Mol. Microbiol*. **94**, 771–793 (2014). 10.1111/mmi.12802 PMC4227967 25243483

[44] Petrova, O. E., Cherny, K. E. & Sauer, K. The diguanylate cyclase GcbA facilitates *Pseudomonas aeruginosa* biofilm dispersion by activating BdlA. *J. Bacteriol*. **197**, 174–187 (2015). 10.1128/JB.02244-14 PMC4288676 25331436

[45] Corley JM, Intile P, Yahr TL (2022). Direct inhibition of RetS synthesis by RsmA contributes to homeostasis of the *Pseudomonas aeruginosa* Gac/Rsm signaling system. J. Bacteriol..

[46] Burrowes E, Baysse C, Adams C, O’Gara F (2006). Influence of the regulatory protein RsmA on cellular functions in *Pseudomonas aeruginosa* PAO1, as revealed by transcriptome analysis. Microbiology.

[47] Ho, Y.-N., Lee, H.-J., Hsieh, C.-T., Peng, C.-C. & Yang, Y.-L. in *Studies in Natural Products Chemistry* (ed. Atta-ur-Rahman) Vol. 59, 431–490 (Elsevier, 2018).

[48] Britigan BE, Rasmussen GT, Cox CD (1997). Augmentation of oxidant injury to human pulmonary epithelial cells by the *Pseudomonas aeruginosa* siderophore pyochelin. Infect. Immun..

[49] Lyczak JB, Cannon CL, Pier GB (2002). Lung infections associated with cystic fibrosis. Clin. Microbiol. Rev..

[50] Sykes, J. E. in *Canine and Feline Infectious Diseases* (ed. Sykes, J. E.) 355–363 (W.B. Saunders, 2014).

[51] Chen L (2020). One-step fabrication of universal slippery lubricated surfaces. Adv. Mater. Interfaces.

[52] MacCallum N (2015). Liquid-infused silicone as a biofouling-free medical material. ACS Biomater. Sci. Eng..

[53] Sotiri I, Overton JC, Waterhouse A, Howell C (2016). Immobilized liquid layers: a new approach to anti-adhesion surfaces for medical applications. Exp. Biol. Med..

[54] Yuan S, Luan S, Yan S, Shi H, Yin J (2015). Facile fabrication of lubricant-infused wrinkling surface for preventing thrombus formation and infection. ACS Appl. Mater. Interfaces.

[55] Li J (2013). Hydrophobic liquid-infused porous polymer surfaces for antibacterial applications. ACS Appl. Mater. Interfaces.

[56] Wang P, Zhang D, Lu Z, Sun S (2016). Fabrication of slippery lubricant-infused porous surface for inhibition of microbially influenced corrosion. ACS Appl. Mater. Interfaces.

[57] Alberts, B. et al. *Molecular Biology of the Cell* (Garland Science, 2017).

[58] Clark, D. P. & Pazdernik, N. J. *Molecular Biology* (Elsevier, 2012).

[59] Lindman B, Medronho B, Alves L, Norgren M, Nordenskiöld L (2021). Hydrophobic interactions control the self-assembly of DNA and cellulose. Q. Rev. Biophys..

[60] Zhao Y, Cieplak M (2017). Proteins at air–water and oil–water interfaces in an all-atom model. Phys. Chem. Chem. Phys..

[61] Beverung CJ, Radke CJ, Blanch HW (1999). Protein adsorption at the oil/water interface: characterization of adsorption kinetics by dynamic interfacial tension measurements. Biophys. Chem..

[62] Mitropoulos V, Mütze A, Fischer P (2014). Mechanical properties of protein adsorption layers at the air/water and oil/water interface: A comparison in light of the thermodynamical stability of proteins. Adv. Colloid Interface Sci..

[63] Bergfreund J, Bertsch P, Kuster S, Fischer P (2018). Effect of oil hydrophobicity on the adsorption and rheology of β-lactoglobulin at oil–water interfaces. Langmuir.

[64] Murray BS (2011). Rheological properties of protein films. COCIS.

[65] Möbius, D. & Miller, R. *Proteins at Liquid Interfaces* (Elsevier, 1998).

[66] Serrien G, Geeraerts G, Ghosh L, Joos P (1992). Dynamic surface properties of adsorbed protein solutions: BSA, casein and buttermilk. Colloids Surf..

[67] Poirier A (2021). Sunflower proteins at air–water and oil–water interfaces. Langmuir.

[68] Howell C, Grinthal A, Sunny S, Aizenberg M, Aizenberg J (2018). Designing liquid-infused surfaces for medical applications: a review. Adv. Mater..

[69] Hong, J. K., Ruhoff, A. M., Mathur, K., Neto, C. & Waterhouse, A. Mechanisms for reduced fibrin clot formation on liquid-infused surfaces. *Adv. Healthc. Mater*. **11**, 2201360 (2022). 10.1002/adhm.202201360 PMC11468711 36040004

[70] Cocco S, Monasson R, Marko JF (2001). Force and kinetic barriers to unzipping of the DNA double helix. Proc. Natl Acad. Sci. USA.

[71] Pingle H (2018). Minimal attachment of *Pseudomonas aeruginosa* to DNA modified surfaces. Biointerphases.

[72] Subbiahdoss G (2019). Antifouling properties of layer by layer DNA coatings. Biofouling.

[73] Ouni OA, Subbiahdoss G, Scheberl A, Reimhult E (2021). DNA polyelectrolyte multilayer coatings are antifouling and promote mammalian cell adhesion. J. Mater..

[74] Barken KB (2008). Roles of type IV pili, flagellum-mediated motility and extracellular DNA in the formation of mature multicellular structures in *Pseudomonas aeruginosa* biofilms. Environ. Microbiol..

[75] Gloag ES (2013). Self-organization of bacterial biofilms is facilitated by extracellular DNA. PNAS.

[76] Whitchurch CB, Tolker-Nielsen T, Ragas PC, Mattick JS (2002). Extracellular DNA required for bacterial biofilm formation. Sci.

[77] Hynen AL (2021). Multiple holins contribute to extracellular DNA release in *Pseudomonas aeruginosa* biofilms. J. Microbiol..

[78] Turnbull L (2016). Explosive cell lysis as a mechanism for the biogenesis of bacterial membrane vesicles and biofilms. Nat. Commun..

[79] Vaccari L (2017). Films of bacteria at interfaces. Adv. Colloid Interface Sci..

[80] Sundin J, Bagheri S (2022). Slip of submerged two-dimensional liquid-infused surfaces in the presence of surfactants. J. Fluid Mech..

[81] Pardhi DS (2022). Microbial surfactants: a journey from fundamentals to recent advances. Front. Microbiol..

[82] Crabbé A (2011). Transcriptional and proteomic responses of *Pseudomonas aeruginosa* PAO1 to spaceflight conditions involve Hfq regulation and reveal a role for oxygen. Appl. Environ. Microbiol..

[83] Chrzanowski Ł, Ławniczak Ł, Czaczyk K (2012). Why do microorganisms produce rhamnolipids?. World J. Microbiol. Biotechnol..

[84] Wilhelm S, Gdynia A, Tielen P, Rosenau F, Jaeger K-E (2007). The autotransporter esterase EstA of *Pseudomonas aeruginosa* is required for rhamnolipid production, cell motility, and biofilm formation. J. Bacteriol..

[85] Zhang L, Hinz AJ, Nadeau J-P, Mah T-F (2011). *Pseudomonas aeruginosa* tssC1 links type VI secretion and biofilm-specific antibiotic resistance. J. Bacteriol..

[86] Heise, C. & Bier, F. F. in *Immobilisation of DNA on Chips II* (ed. Wittmann, C.) 1–25 (Springer, 2005).

[87] Nimse SB, Song K, Sonawane MD, Sayyed DR, Kim T (2014). Immobilization techniques for microarray: challenges and applications. J. Sens..

[88] De Kievit TR, Gillis R, Marx S, Brown C, Iglewski BH (2001). Quorum-sensing genes in *Pseudomonas aeruginosa* biofilms: their role and expression patterns. Appl. Environ. Microbiol..

[89] Horneck G, Klaus DM, Mancinelli RL (2010). Space microbiology. Microbiol. Mol. Biol. Rev..

[90] Heydorn A (2000). Quantification of biofilm structures by the novel computer program COMSTAT. J. Microbiol..

[91] Heydorn, A. & Ersbøll, B. K. Comstat 2. *Welcome to the Comstat 2 homepage.* http://www.comstat.dk/ (2015).

[92] Vorregaard, M. *Comstat2—A Modern 3D Image Analysis Environment for Biofilms* (Technical university of Denmark (DTU), (2008).

[93] Andrews, S. *FastQC A. Quality Control tool for High Throughput Sequence Data* (2010).

[94] Ewels P, Magnusson M, Lundin S, Käller M (2016). MultiQC: summarize analysis results for multiple tools and samples in a single report. J. Bioinform..

[95] Martin M (2011). Cutadapt removes adapter sequences from high-throughput sequencing reads. EMBnet J..

[96] Langmead B, Salzberg SL (2012). Fast gapped-read alignment with Bowtie 2. Nat. Methods.

[97] Danecek P (2021). Twelve years of SAMtools and BCFtools. GigaScience.

[98] Liao Y, Smyth GK, Shi W (2019). The R package Rsubread is easier, faster, cheaper and better for alignment and quantification of RNA sequencing reads. Nucleic Acids Res..

[99] R Core Team. R: The R Project for Statistical Computing. (2022).

[100] Love MI, Huber W, Anders S (2014). Moderated estimation of fold change and dispersion for RNA-seq data with DESeq2. Genome Biol..

[101] Koeppen, K., Hampton, T. H., Neff, S. L., & Stanton, B. A. ESKAPE Act Plus: pathway activation analysis for bacterial pathogens. *mSystems*10.1128/msystems.00468-22 (2022). 10.1128/msystems.00468-22 PMC9764987 36259735

[102] Zea, L. & Flores, P. *Characterization of Biofilm Formation, Growth, and Gene Expression on Different Materials and Environmental Conditions in Microgravity - Morphology Data*. 10.26030/bp7m-0f62 (2023).

[103] Zea, L. & Flores, P. *Characterization of Biofilm Formation, Growth, and Gene Expression on Different Materials and Environmental Conditions in Microgravity - Gene Expression Data*. 10.26030/d5dg-7s68 (2023).

