# Supplementary material for: Biofilm formation of Pseudomonas aeruginosa in spaceflight is minimized on lubricant impregnated surfaces
Source: NPJ Microgravity. 2023 Aug 16;9:66. doi: 10.1038/s41526-023-00316-w (PMC10432549; doi:10.1038/s41526-023-00316-w)
Supplement: Supplementary file 1 — Supplemental Information [file 41526_2023_316_MOESM1_ESM.pdf]

Supplementary Information

Supplementary Figures

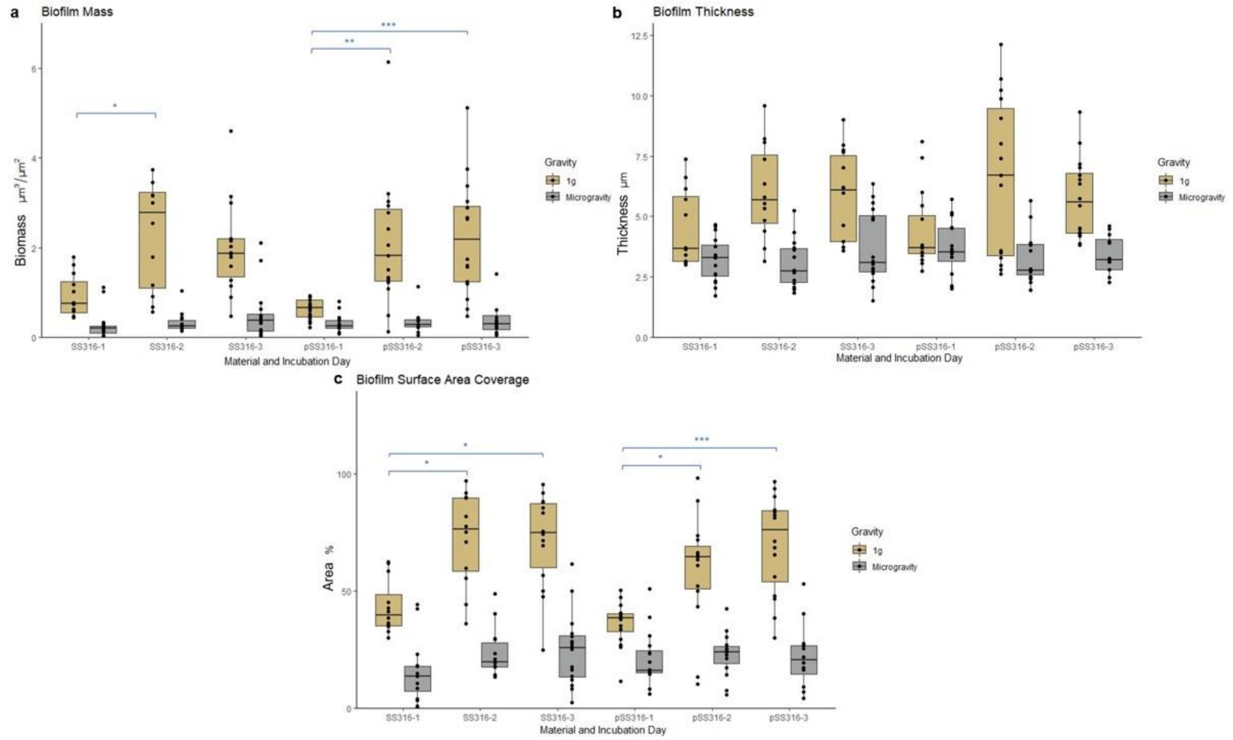

Supplementary Figure 1. Passivation of SS316 has no significant effect on PA14 biofilm morphology. Box plots of (**a**) biofilm mass, (**b**) thickness, and (**c**) surface area coverage as a function of time (1, 2, and 3-day old biofilms) and gravitational condition. Statistical significance specified with horizontal brackets for differences between time points (blue brackets). Significance of differences between ground and microgravity samples are not specified in the plot as their not relevant for the decision of merging SS316 and pSS316 data sets. SS316= stainless steel. pSS316 = passivated stainless steel. n = 4 biological replicates each imaged in 4 fields of view. Dunn’s test with Bonferroni correction ***$p \leq 0.001$, ** $p \leq 0.01$, *$p \leq 0.05$. Box central line= median, bounds of box=first and third quartile, whiskers=minimum and maximum values (without outliers).

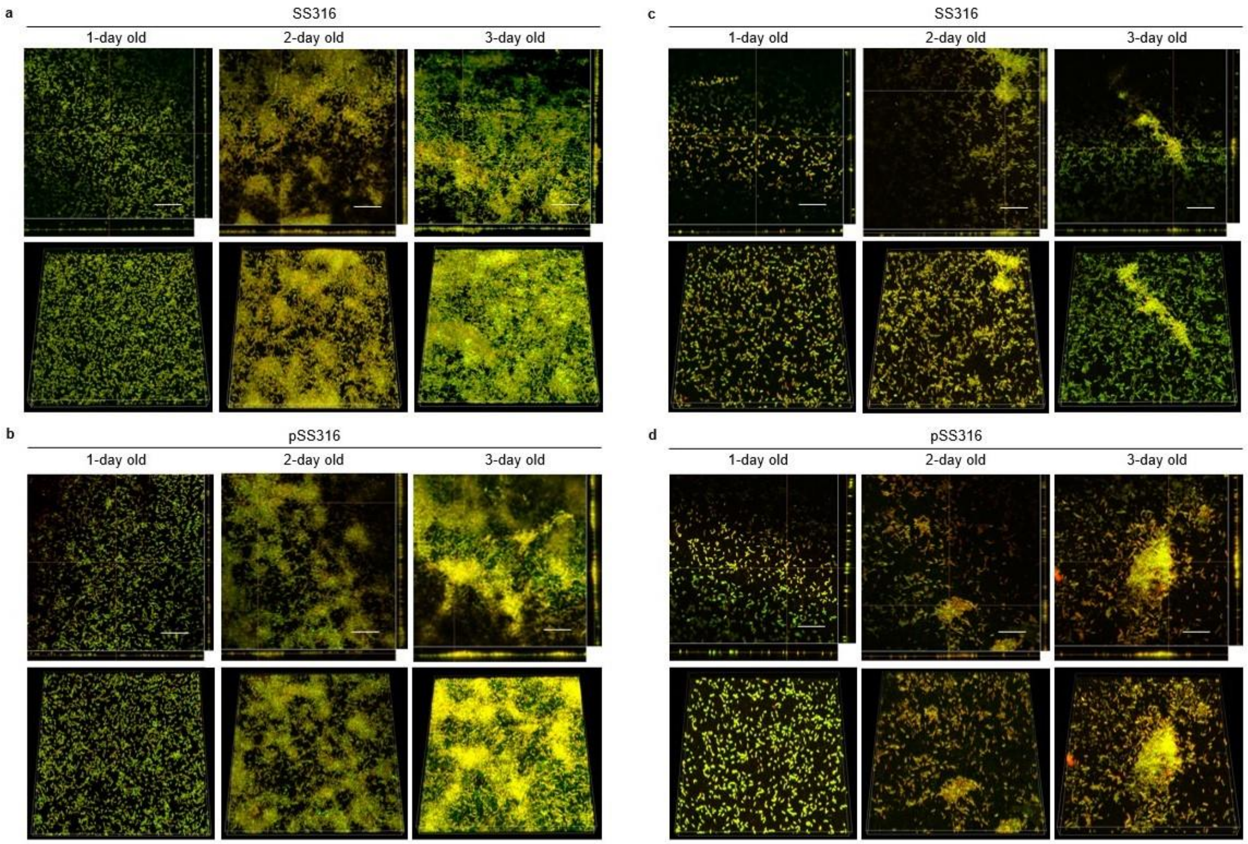

Supplementary Figure 2. Passivation of SS316 does not affect PA14 biofilm morphology. Representative confocal microscopy images of 1, 2, and 3-day old biofilms formed over SS316 and pSS316. Nucleic acids were stained in red and lipids were stained in green. First row per material is a bottom slice image with side-view panels of a cross-section point in the biofilm (specific section marked by the orange lines). Second row per material is a volume view of the complete z-stack image. Images correspond to biofilms grown in 1 *g* over (**a**) SS and (**b**) LIS, or biofilms grown in microgravity over (**c**) SS and (**d**) LIS. Scale bars are 20 μm. Microscopy images, both in microgravity and ground, show similar biofilm morphology on both materials.

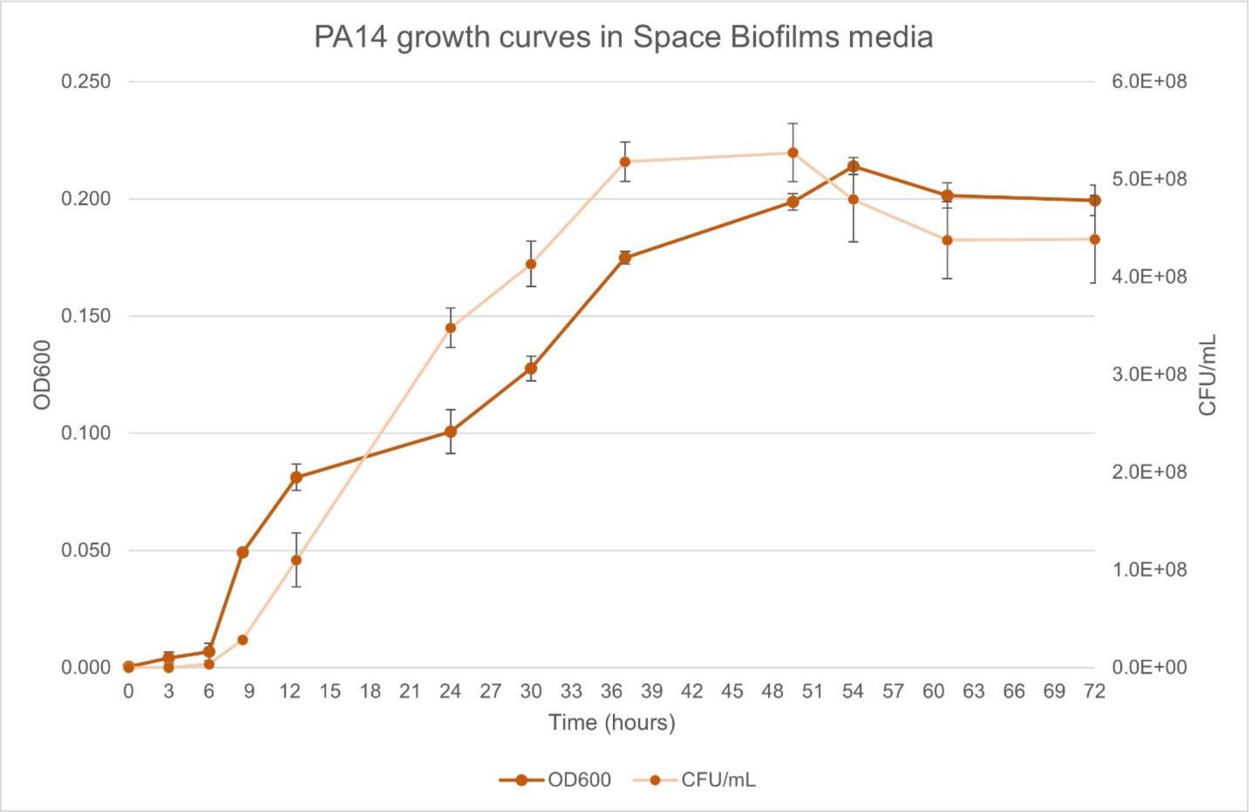

Supplementary Figure 3. *Pseudomonas aeruginosa* PA14 liquid turbidity correlated to viable cell count. Growth curve of PA14 measuring turbidity (OD_{600}) and viable planktonic cells (CFU/mL) of the liquid culture as seen at 1 *g*. Error bars represent the standard deviation (s.d.). n = 3 biological replicates. the Pseudomonas aeruginosa PA14 strain used in our experiment. The OD_{600} measurements of *P. aeruginosa* PA14 in our experimental configuration allow correct distinction of the bacterial stages of growth, and OD_{600} measurements are also correlated to the viable cell density.

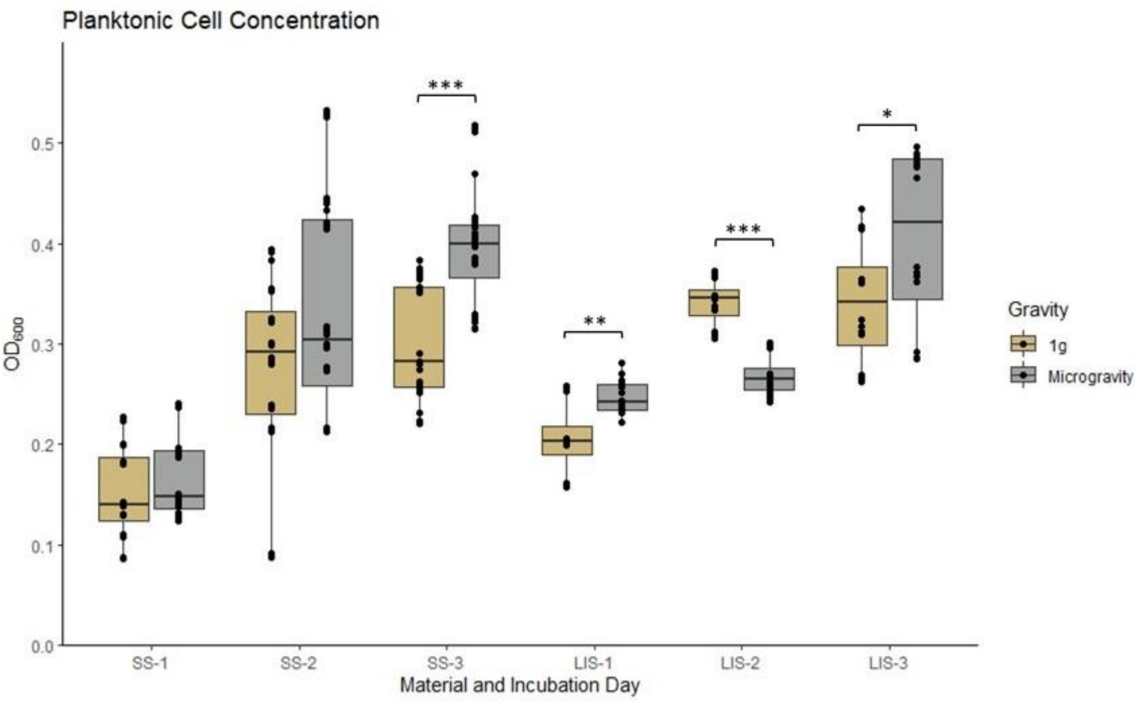

Supplementary Figure 4. *Pseudomonas aeruginosa* Planktonic cell OD. Concentration of planktonic cells plotted as a function of time (1, 2, and 3-day old biofilms) and gravitational condition. Statistical significance specified with horizontal brackets for differences between gravitational regimes. SS= stainless steel. LIS = lubricant impregnated surface. For SS, n = 8 biological replicates each with 4 technical replicates. For LIS, n = 4 biological replicates each with 4 technical replicates. Dunn's test with Bonferroni correction ***p≤0.001, ** p≤0.01, *p≤0.05. Box central line= median, bounds of box=first and third quartile, whiskers=minimum and maximum values (without outliers).

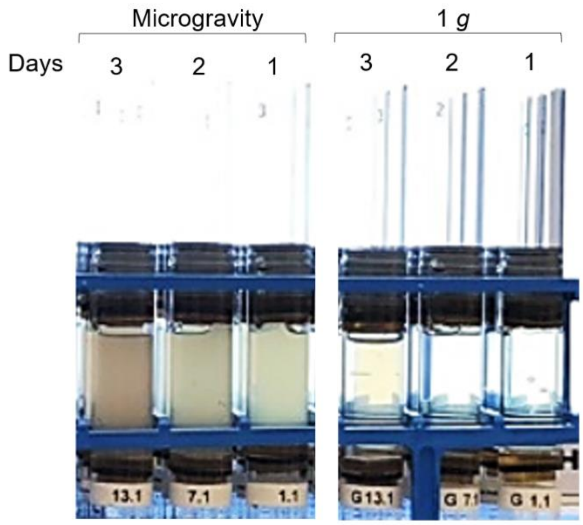

Supplementary Figure 5. Visual comparison of FPAs in microgravity vs 1*g*. Four FPAs per condition stacked in front of each other to increase color contrast of the liquid culture. Besides the difference in color, the difference in turbidity of samples in microgravity, with respect to samples at 1*g*, can also be appreciated.

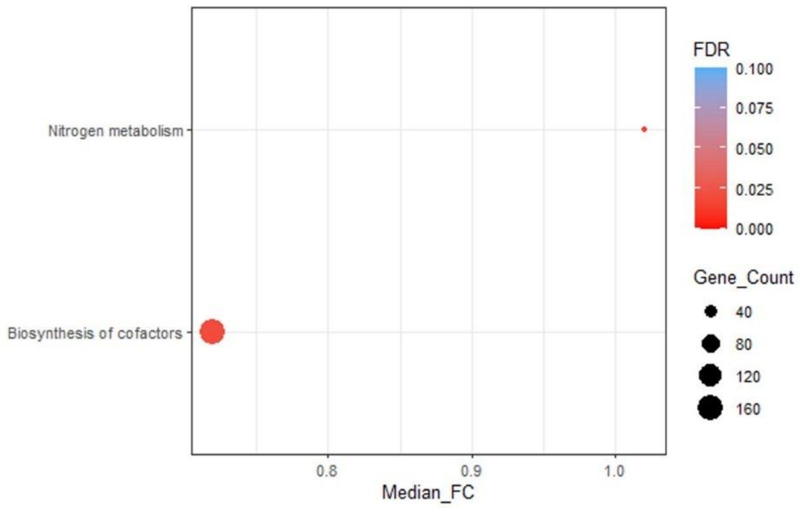

Supplementary Figure 6. Pathways enrichment analysis of *P. aeruginosa* biofilms formed on SS in space with respect to Earth. KEGG pathways enrichment analysis from 3-day-old biofilms grown on stainless steel coupons in microgravity with respect to 1 *g*. n = 7 biological replicates per gravitational condition.

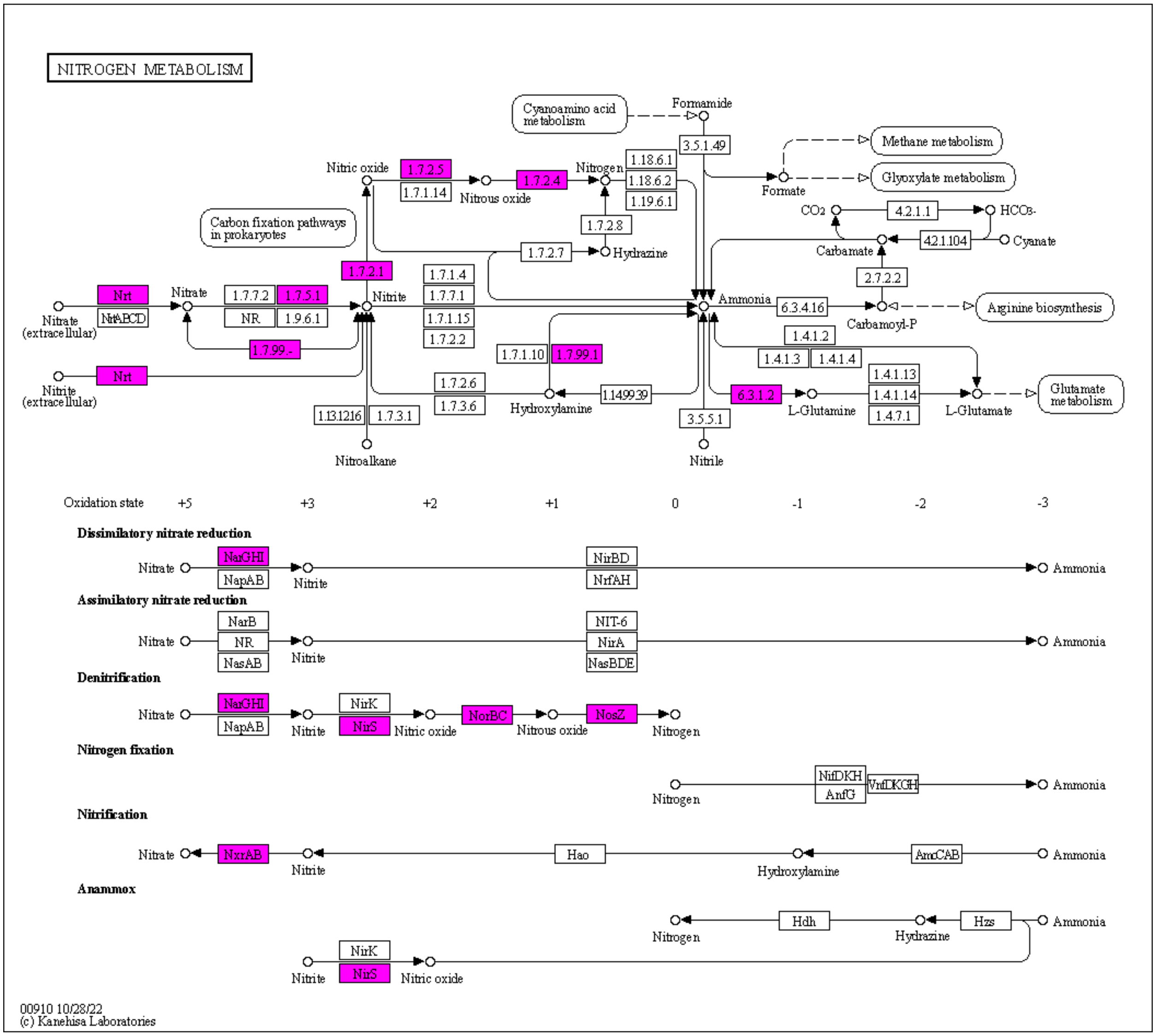

Supplementary Figure 7. Enrichment of nitrogen metabolism pathway in biofilms grown over SS in microgravity. Pathway topology with gene boxes in pink that represent the genes with increased expression in microgravity. Generated with KEGG Mapper.

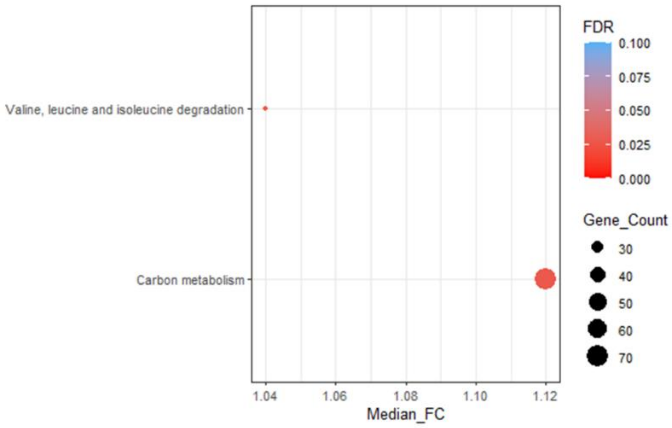

Supplementary Figure 8. Pathways enrichment analysis of *P. aeruginosa* 3-day-old biofilms formed on LIS with respect to SS on Earth. KEGG pathways enrichment analysis from 3-day-old biofilms grown on LIS coupons with respect to SS biofilms on Earth. For SS, n = 7 biological replicates. For LIS, n =4 biological replicates.

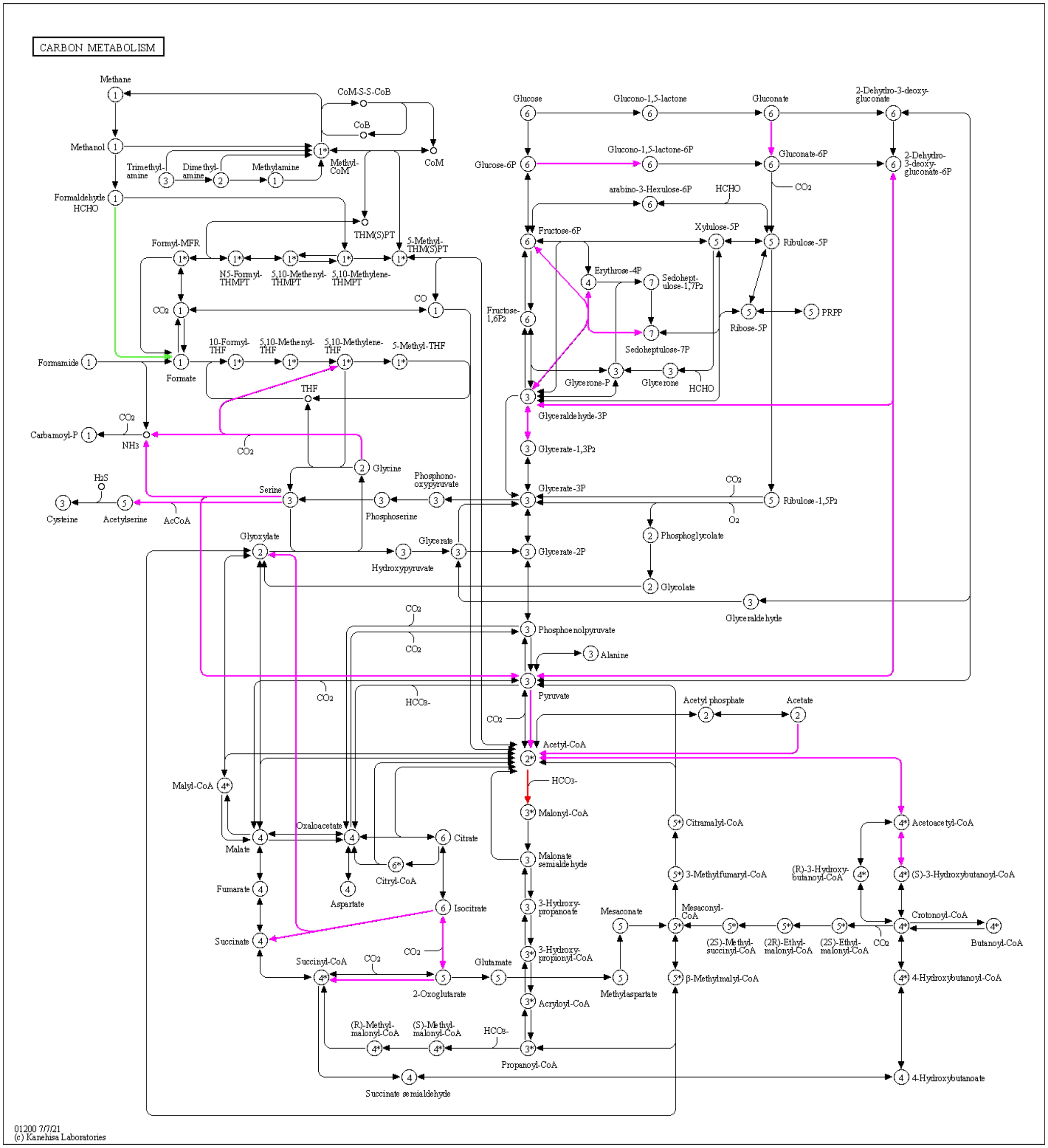

Supplementary Figure 9. Enrichment of carbon metabolism pathway in biofilms grown over LIS with respect to biofilms on SS on Earth. Pathway topology with lines in pink and green that represent the genes with increased and decreased expression in microgravity, respectively. Generated with KEGG Mapper.

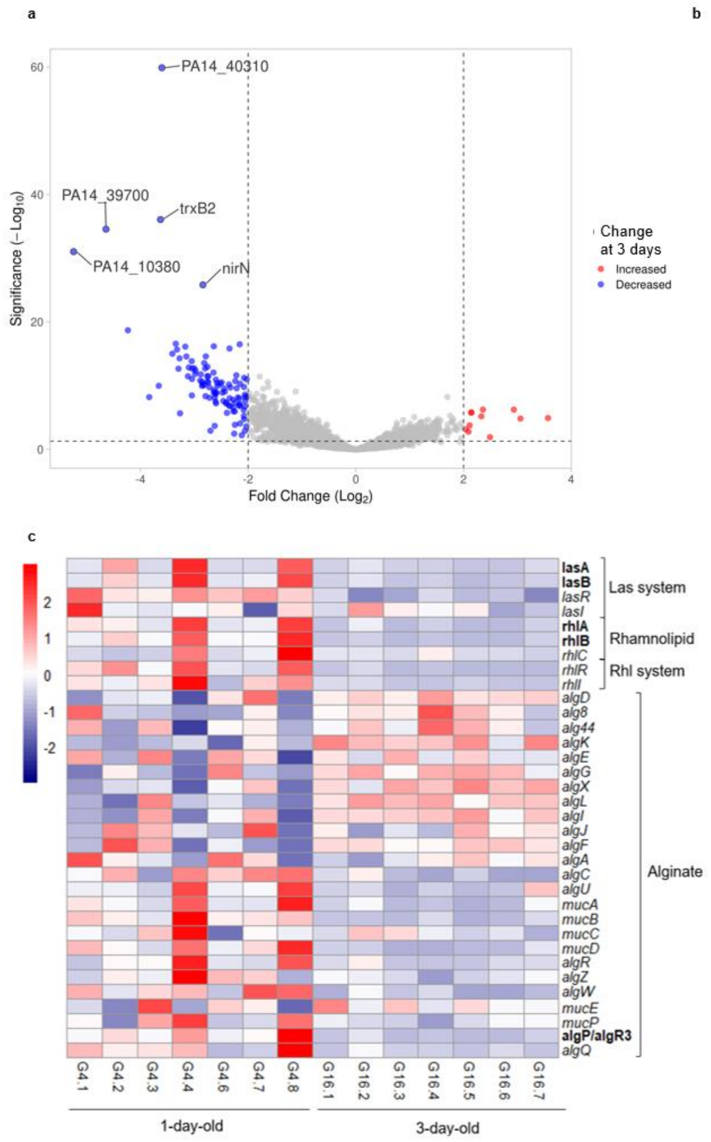

Supplementary Figure 10. Transcriptional differences between 3-day old *P. aeruginosa* biofilms formed on SS with respect to 1-day old biofilms, on Earth. (**a**) Volcano plot and (**b**) MA plot of differential gene expression. (**c**) Heatmap of normalized counts for relevant genes involved in *P. aeruginosa* virulome, significant differentially expressed genes with fold change ≥2 in bold. n = 7 biological replicates per condition.

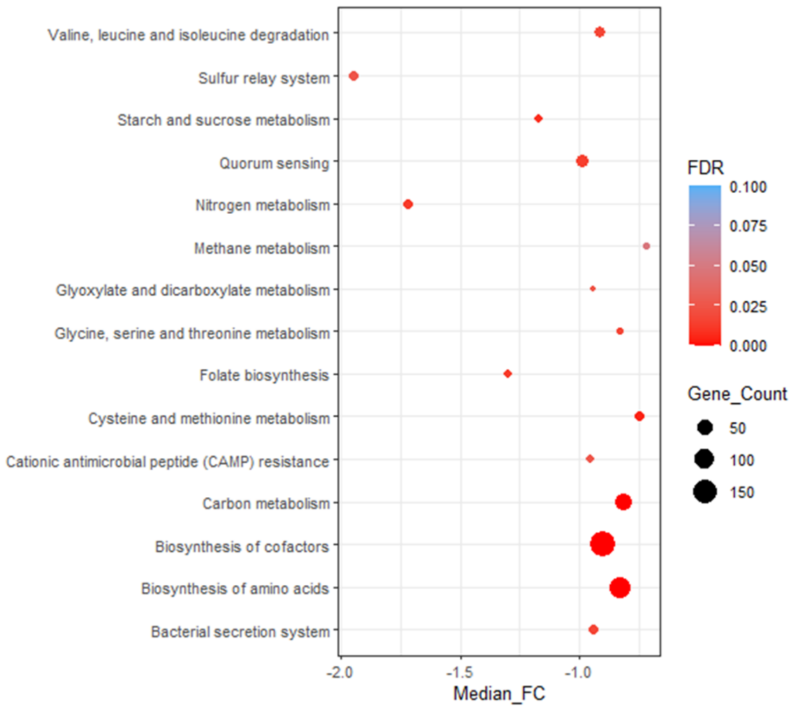

Supplementary Figure 11. Pathways enrichment analysis of *P. aeruginosa* 3-day-old biofilms with respect to 1-day-old biofilms grown over SS on Earth. KEGG pathways enrichment analysis from 3-day-old biofilms compared to 1-day-old biofilms grown on SS coupons on Earth. n = 7 biological replicates.

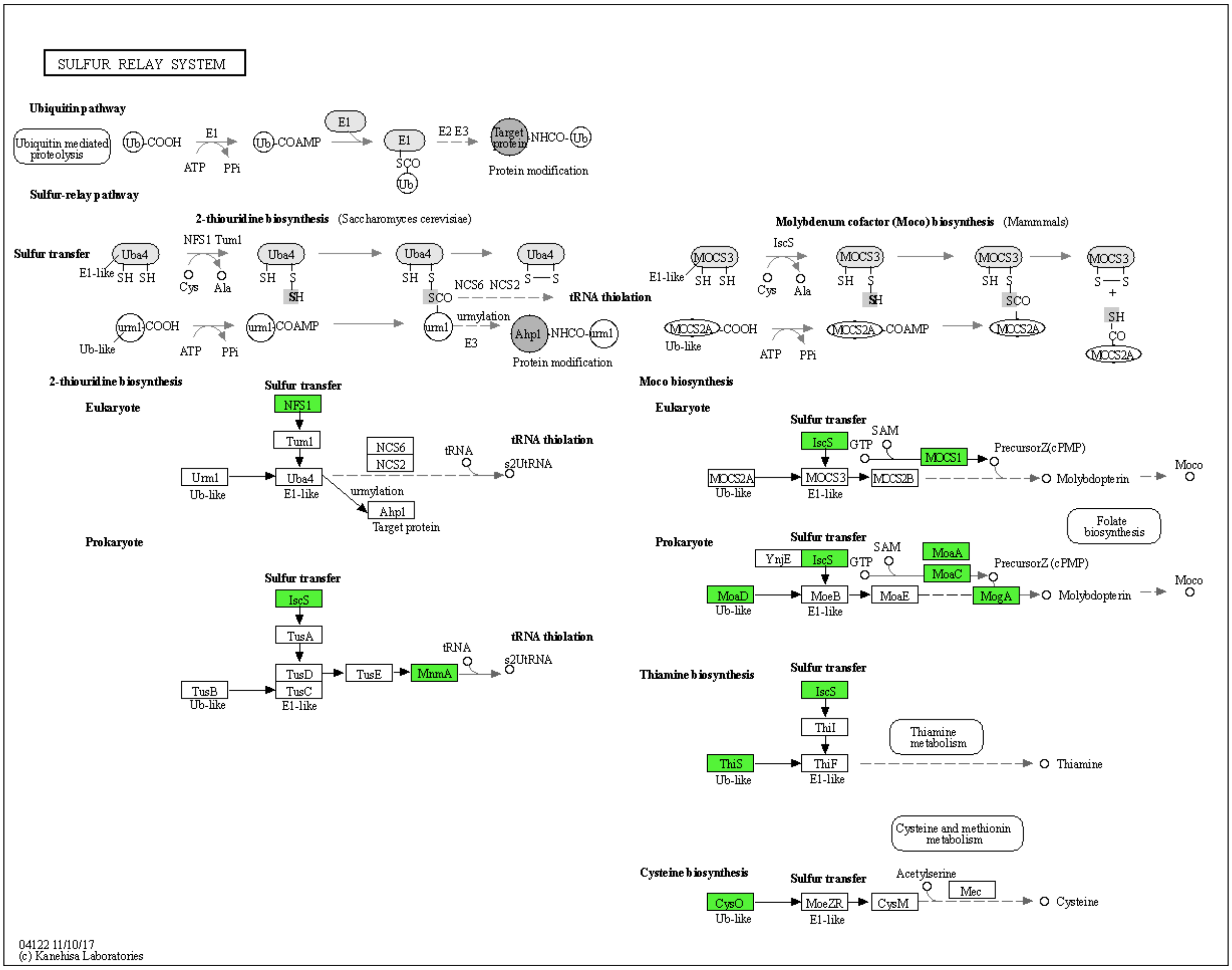

Supplementary Figure 12. Enrichment of sulfur relay system pathway in biofilms grown over LIS with respect to biofilms on SS on Earth. Pathway topology with gene boxes in green that represent the genes with decreased expression in microgravity at 3 days of incubation. Generated with KEGG Mapper.

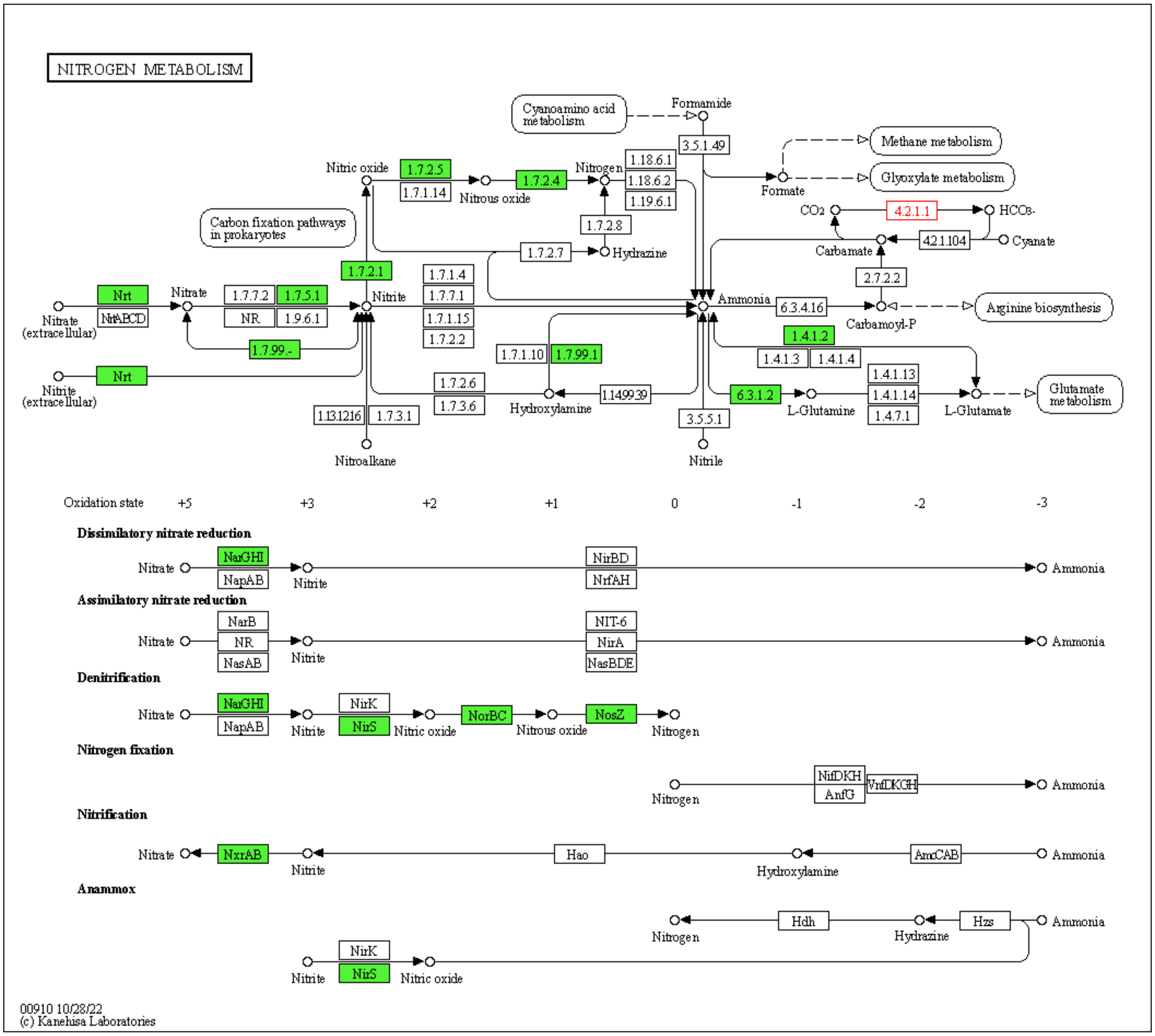

Supplementary Figure 13. Enrichment of nitrogen metabolism pathway in biofilms grown over LIS with respect to biofilms on SS on Earth. Pathway topology with gene boxes in green that represent the genes with decreased expression in microgravity at 3 days of incubation. Generated with KEGG Mapper.

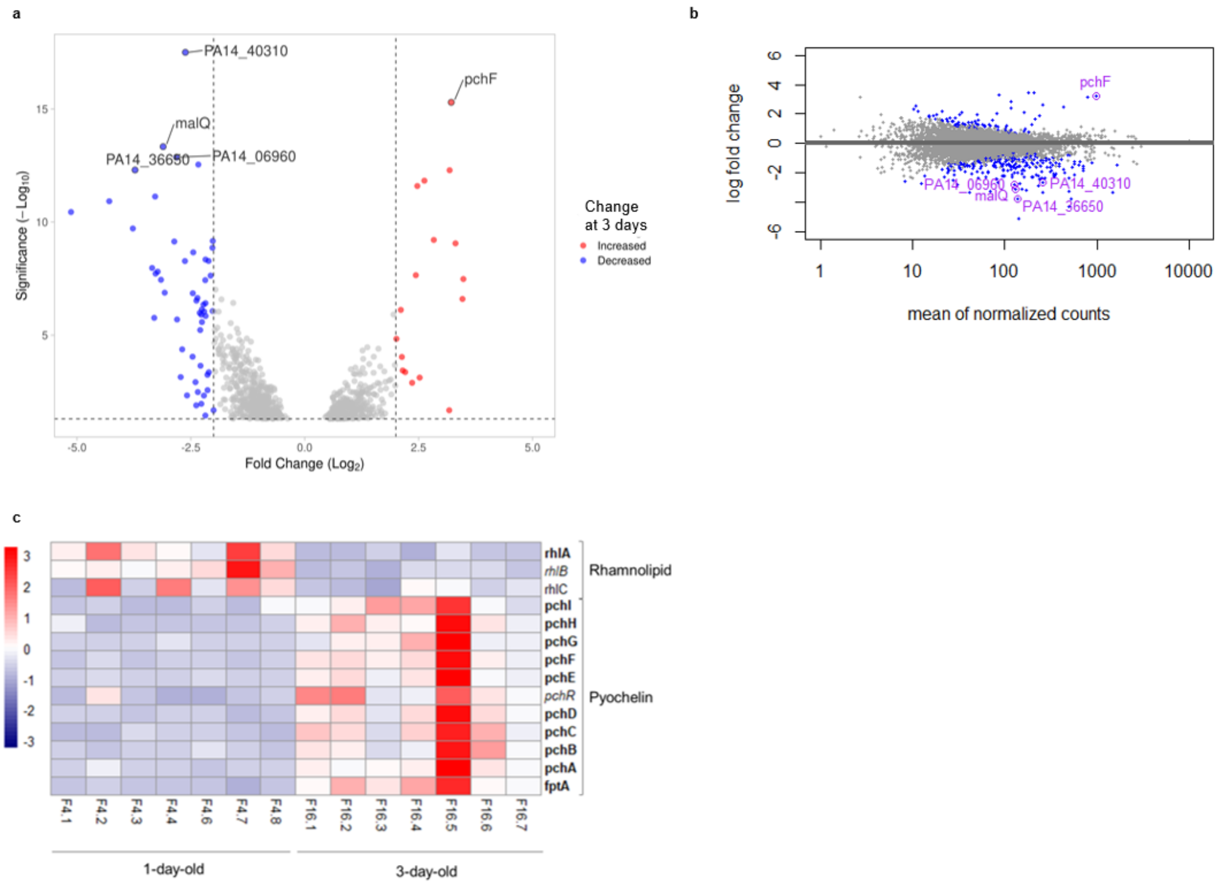

Supplementary Figure 14. Transcriptional differences between 3-day old *P. aeruginosa* biofilms formed on SS with respect to 1-day old biofilms, in microgravity. (**a**) Volcano plot and (**b**) MA plot of differential gene expression. (**c**) Heatmap of normalized counts for relevant genes involved in *P. aeruginosa* virulome, significant differentially expressed genes with fold change ≥2 in bold. n = 7 biological replicates per condition.

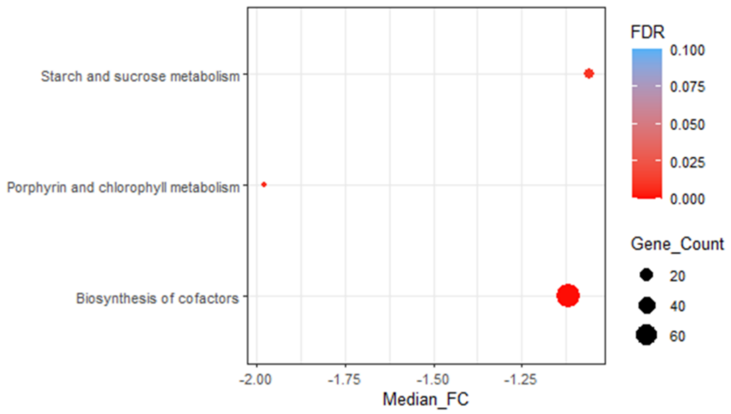

Supplementary Figure 15. Pathways enrichment analysis of *P. aeruginosa* 3-day-old biofilms with respect to 1-day-old biofilms grown over SS in space. KEGG pathways enrichment analysis from 3-day-old biofilms compared to 1-day-old biofilms grown on SS coupons in microgravity. n = 7 biological replicates.

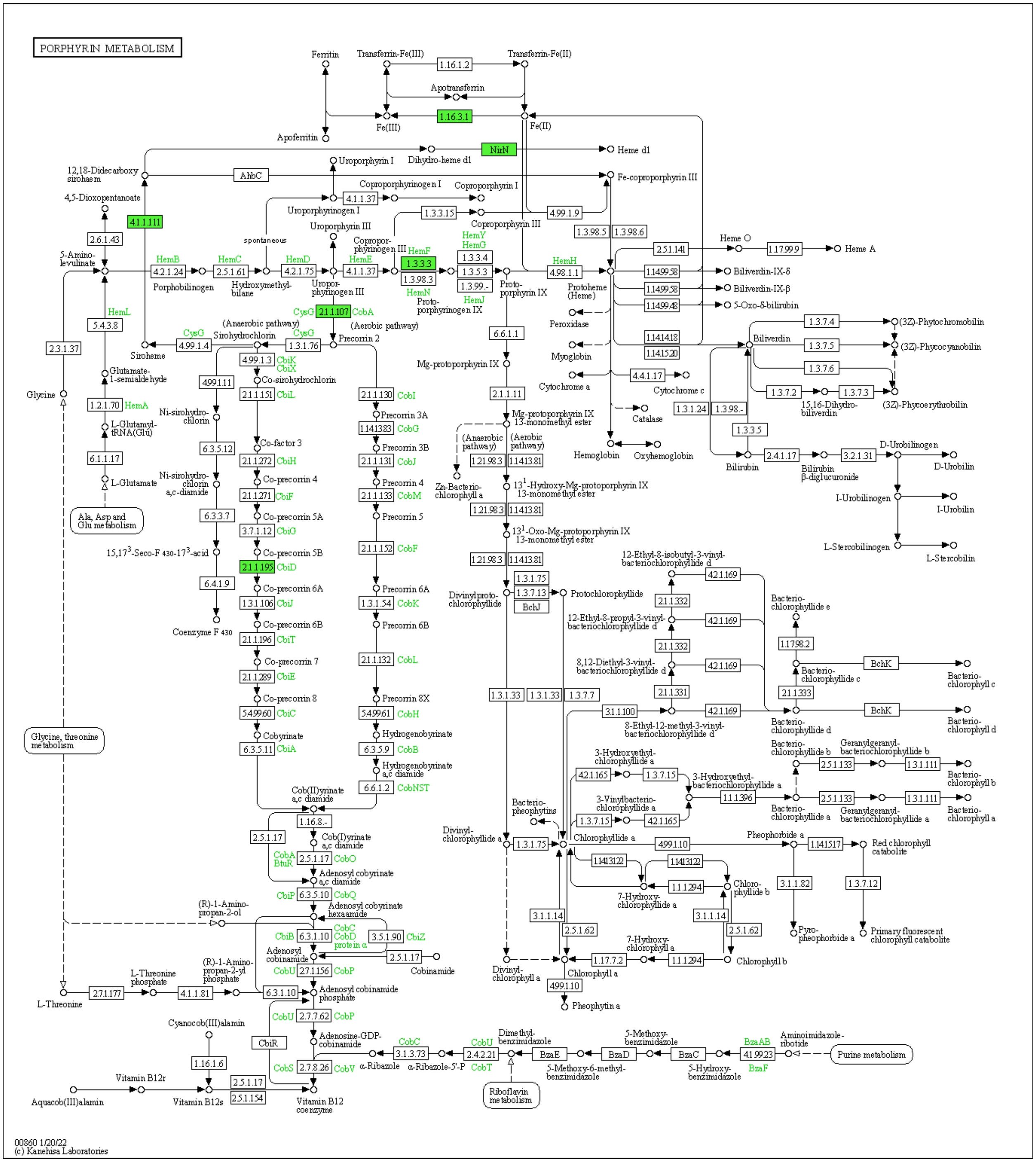

Supplementary Figure 16. Enrichment of pathway in 3-dayold biofilms grown over SS in space. Pathway topology with gene boxes in green that represent the genes with decreased expression at 3 days of incubation with respect to 1-day of incubation. Generated with KEGG Mapper.

Supplementary Tables

Supplementary Table 12. Quality results (RIN values) for the RNA extracted from biofilm ground and spaceflight samples

Material	ID	Incubation Day	Gravity	RIN	ID	Incubation Day	Gravity	RIN
**SS316**	G4.1	1	Ground	8.7	G16.1	3	Ground	NA
**SS316**	G4.2	1	Ground	9.2	G16.2	3	Ground	NA
**SS316**	G4.3	1	Ground	8.1	G16.3	3	Ground	6.4
**SS316**	G4.4	1	Ground	NA	G16.4	3	Ground	8
**pSS316**	G4.6	1	Ground	7.6	G16.5	3	Ground	NA
**pSS316**	G4.7	1	Ground	NA	G16.6	3	Ground	NA
**pSS316**	G4.8	1	Ground	NA	G16.7	3	Ground	NA
**LIS**	G5.1	1	Ground	NA	G17.1	3	Ground	NA
**LIS**	G5.2	1	Ground	NA	G17.2	3	Ground	NA
**LIS**	G5.3	1	Ground	NA	G17.3	3	Ground	NA
**LIS**	G.5.4	1	Ground	NA	G17.4	3	Ground	NA
**SS316**	F4.1	1	Microgravity	NA	F16.1	3	Microgravity	9.1
**SS316**	F4.2	1	Microgravity	NA	F16.2	3	Microgravity	9.2
**SS316**	F4.3	1	Microgravity	NA	F16.3	3	Microgravity	7
**SS316**	F4.4	1	Microgravity	NA	F16.4	3	Microgravity	NA
**pSS316**	F4.6	1	Microgravity	NA	F16.5	3	Microgravity	7.8
**pSS316**	F4.7	1	Microgravity	NA	F16.6	3	Microgravity	NA
**pSS316**	F4.8	1	Microgravity	9.2	F16.7	3	Microgravity	NA
**LIS**	F5.1	1	Microgravity	8.1	F17.1	3	Microgravity	NA
**LIS**	F5.2	1	Microgravity	9.2	F17.2	3	Microgravity	NA
**LIS**	F5.3	1	Microgravity	NA	F17.3	3	Microgravity	7.5
**LIS**	F.5.4	1	Microgravity	9.6	F17.4	3	Microgravity	NA

NA= data not available. Unfortunately, the RIN values of the samples were lost due to a faulty equipment at the BioFrontiers Sequencing Core. The RIN values presented in Table S12 correspond to the samples that had enough volume left to be run a second time.

The following tables are not included in this PDF file due to their large size but can be found as individual excel files downloadable in the Supplementary Information section of Nature Microgravity's paper website:

Supplementary Table 1. Raw data for box plots of Supplementary Figure 1

Supplementary Table 2. DEG list of biofilms grown on pSS316 with respect to SS316 on microgravity day 2

Supplementary Table 3. Raw data for OD600 plot of Supplementary Figure 4

Supplementary Table 4. Raw data of Biofilm Biomass, Thickness, and Surface Coverage

Supplementary Table 5. DEG list and normalized read counts per sample of biofilms grown in microgravity with respect to 1*g* for SS day 3

Supplementary Table 6. DEG list and normalized read counts per sample of biofilms grown in microgravity with respect to 1*g* for LIS day 3

Supplementary Table 7. DEG list and normalized read counts per sample of biofilms grown on LIS with respect to SS for ground controls day 3

Supplementary Table 8. DEG list and normalized read counts per sample of biofilms grown on LIS with respect to SS in microgravity day 3

Supplementary Table 9. Shared DEG in both gravitational regimes of biofilms on LIS with respect to SS at day 3

Supplementary Table 10. DEG list and normalized read counts per sample of 3-day-old with respect to 1-day-old biofilms on SS in 1*g*

Supplementary Table 11. DEG list and normalized read counts per sample of 3-day-old with respect to 1-day-old biofilms on SS in microgravity

Supplementary Table 13. Additional transcriptomic information
