# Supplementary material for: Biofilm formation of Pseudomonas aeruginosa in spaceflight is minimized on lubricant impregnated surfaces
Source: NPJ Microgravity. 2023 Aug 16;9:66. doi: 10.1038/s41526-023-00316-w (PMC10432549; doi:10.1038/s41526-023-00316-w)
Supplement: Supplementary file 14 — Reporting Summary [file 41526_2023_316_MOESM14_ESM.pdf]

nature portfolio

Corresponding author(s): Luis Zea, Kripa Varanasi, Pamela Flores

Last updated by author(s): Jul 13, 2023

Reporting Summary

Nature Portfolio wishes to improve the reproducibility of the work that we publish. This form provides structure for consistency and transparency in reporting. For further information on Nature Portfolio policies, see our Editorial Policies and the Editorial Policy Checklist.

Statistics

For all statistical analyses, confirm that the following items are present in the figure legend, table legend, main text, or Methods section.

n/a	Confirmed	
☐	☒	The exact sample size (n) for each experimental group/condition, given as a discrete number and unit of measurement
☐	☒	A statement on whether measurements were taken from distinct samples or whether the same sample was measured repeatedly
☐	☒	The statistical test(s) used AND whether they are one- or two-sided<br>*Only common tests should be described solely by name; describe more complex techniques in the Methods section.*
☐	☒	A description of all covariates tested
☐	☒	A description of any assumptions or corrections, such as tests of normality and adjustment for multiple comparisons
☐	☒	A full description of the statistical parameters including central tendency (e.g. means) or other basic estimates (e.g. regression coefficient) AND variation (e.g. standard deviation) or associated estimates of uncertainty (e.g. confidence intervals)
☐	☒	For null hypothesis testing, the test statistic (e.g. F, t, r) with confidence intervals, effect sizes, degrees of freedom and P value noted<br>*Give P values as exact values whenever suitable.*
☒	☐	For Bayesian analysis, information on the choice of priors and Markov chain Monte Carlo settings
☒	☐	For hierarchical and complex designs, identification of the appropriate level for tests and full reporting of outcomes
☒	☐	Estimates of effect sizes (e.g. Cohen's d, Pearson's r), indicating how they were calculated

Our web collection on statistics for biologists contains articles on many of the points above.

Software and code

Policy information about availability of computer code

Data collection	The publicly available COMSTAT2 software was used to calculate biofilm mass (µm3/µm2), thickness (µm), and surface area coverage (%) of the biofilms with automatic thresholding.<br>For the transcriptomic data, raw sequencing files were checked for quality control using FastQC (version 0.11.9) and multiQC (version 1.0.dev0). Then Illumina adapter contamination was eliminated using Trimgalore (version 0.6.6) with cutadapt (version 2.6), after which quality control was performed again. Trimmed reads were mapped to PA14 rRNA sequences to remove the contamination, the resulting unmapped reads were then mapped to the Ensembl Bacteria reference genome of Pseudomonas aeruginosa UCBPP-PA14 (accession number GCA_000014625), both mapping steps were performed using Bowtie2 (version 2.4.4). The mapping results were sorted, indexed, and converted from .sam files to .bam files using SAMtools (version 1.11). The reads per gene were counted with Feature Counts (Rsubread package version 2.8.2).
Data analysis	Biofilm biomass, thickness, and surface area coverage data was compared between groups using two-sided Kruskal Wallis and Dunn's (with Bonferroni correction) tests in R to determine if differences observed were significant.<br>The differential gene expression analyses were done in an R (version 4.1.1) with DESeq2 (version 1.34.0). Pathways enrichment analyses were performed online using ESKAPE Act PLUS101 and the whole list of DEG (including ≤2-fold change genes) per condition as input.

For manuscripts utilizing custom algorithms or software that are central to the research but not yet described in published literature, software must be made available to editors and reviewers. We strongly encourage code deposition in a community repository (e.g. GitHub). See the Nature Portfolio guidelines for submitting code & software for further information.

Data

Policy information about availability of data

All manuscripts must include a data availability statement. This statement should provide the following information, where applicable:

- Accession codes, unique identifiers, or web links for publicly available datasets
- A description of any restrictions on data availability
- For clinical datasets or third party data, please ensure that the statement adheres to our policy

Data available on NASA's Open Science Data Repository (OSDR, https://osdr.nasa.gov/bio/). The microscopy data under study OSD-627 and DOI: 10.26030/bp7m-0f62102. The transcriptomic data under study OSD-554 DOI: 10.26030/d5dg-7s68103. The data will also be available on NASA's Physical Sciences Informatics (PSI) data repository (https://psi.nasa.gov).

Research involving human participants, their data, or biological material

Policy information about studies with human participants or human data. See also policy information about sex, gender (identity/presentation), and sexual orientation and race, ethnicity and racism.

Reporting on sex and gender	This information was not collected. No human research was performed.
Reporting on race, ethnicity, or other socially relevant groupings	This information was not collected. No human research was performed.
Population characteristics	This information was not collected. No human research was performed.
Recruitment	This information was not collected. No human research was performed.
Ethics oversight	This information was not collected. No human research was performed.

Note that full information on the approval of the study protocol must also be provided in the manuscript.

Field-specific reporting

Please select the one below that is the best fit for your research. If you are not sure, read the appropriate sections before making your selection.

☒ Life sciences ☐ Behavioural & social sciences ☐ Ecological, evolutionary & environmental sciences

For a reference copy of the document with all sections, see nature.com/documents/nr-reporting-summary-flat.pdf

Life sciences study design

All studies must disclose on these points even when the disclosure is negative.

Sample size	This is a continuous endpoint, independent samples study. Based on previous simulated microgravity data we estimated that the least effect size of microgravity will be a 20% increase or decrease in biomass, thickness, and surface area coverage. Using the ClinCalc Sample Size Online Calculator (https://clincalc.com/stats/samplesize.aspx) with β =0.2 (type II error), α=0.05 (type I error) and a power of 0.8, we obtained a sample size of four for each condition. This sample size was used to determine the number of biological replicates to use in our biofilm conditions. For gene expression analysis the power is greater and the effect size too, and our sample size of four and seven replicates per condition is more than enough to find significant changes in gene expression.
Data exclusions	The transcriptomic reads corresponding to rRNA were excluded from the analysis by computational removal. This was done prior to performing the gene expression analysis, as rRNA was supposed to be removed before sequencing, but the rRNA contamination was not removed properly with the sequencing kit used.
Replication	All experiments were performed with the same flight hardware, including ground controls. For morphology samples, the microscopy images were taken in the center of the coupon to avoid any user bias. All attempts at replication were successful.
Randomization	Allocation was not randomized as all samples were prepared with the same initial inoculum and exposed to same conditions. The hardware does not create a gradient of temperature that would affect samples differently.
Blinding	Investigators were blinded to gravitational condition and incubation time of samples being imaged, but the material is easily distinguished by eye sight so it was not possible to blind to material surface being tested.

Reporting for specific materials, systems and methods

We require information from authors about some types of materials, experimental systems and methods used in many studies. Here, indicate whether each material, system or method listed is relevant to your study. If you are not sure if a list item applies to your research, read the appropriate section before selecting a response.

Materials & experimental systems

n/a	Involved in the study
☒	☐ Antibodies
☒	☐ Eukaryotic cell lines
☒	☐ Palaeontology and archaeology
☒	☐ Animals and other organisms
☒	☐ Clinical data
☒	☐ Dual use research of concern
☒	☐ Plants

Methods

n/a	Involved in the study
☒	☐ ChIP-seq
☒	☐ Flow cytometry
☒	☐ MRI-based neuroimaging
